# Beyond weight loss, cognitive health, and glycemic control: taurine supplementation as a reprogrammer of adipose tissue plasticity, physical performance, metabolic flexibility, neuroinflammaging, and cardiac remodeling in obesity, aging, and diabetes

**DOI:** 10.3389/fnut.2026.1783074

**Published:** 2026-03-26

**Authors:** LiMing Zhang, Yao Zhang, Yu Wang

**Affiliations:** 1 School of Sports Science and Technology, Bangkokthonburi University, Bangkok, Thailand; 2 Institute of Physical Education, Harbin Preschool Teachers College, Harbin, Heilongjiang, China; 3 Independent Researcher, Milwaukee, WI, United States

**Keywords:** aging, diabetes, exercise, obesity, taurine

## Abstract

Obesity, senescence, and diabetes mellitus represent interrelated conditions defined by compromised metabolic adaptability, persistent low-grade inflammation, dysfunction of adipose tissue, and a gradual deterioration in physical, cardiovascular, and cognitive capabilities. Conventional therapeutic approaches have predominantly emphasized weight reduction and glycemic regulation; however, these methodologies frequently neglect to consider the intricate multisystem pathophysiology that underpins the progression of these diseases. This review collates the prevailing experimental and clinical insights illustrating that the additive effects of taurine and exercise foster adipose tissue adaptability, augment mitochondrial functionality, enhance insulin sensitivity, and bolster physical performance as well as metabolic flexibility. Concurrently, taurine and physical exercise intersect at critical cardioprotective mechanisms, encompassing the modulation of apoptotic pathways, the regulation of extracellular matrix remodeling, and the activation of PI3K/Akt signaling, consequently mitigating maladaptive cardiac remodeling linked to obesity, aging, and diabetes mellitus. By synthesizing evidence from metabolic, cardiovascular, and neurophysiological frameworks, this review elucidates taurine supplementation in conjunction with physical exercise as a non-pharmacological intervention that transcends traditional therapeutic targets, thereby providing a multifaceted strategy to enhance systemic health and functional capacity in the context of metabolic and age-related disorders.

## 1 Introduction

Obesity, senescence, and diabetes represent a triadic constellation of health challenges that are increasingly prevalent and interrelated, imposing significant metabolic, cardiovascular, and neurological burdens on a global scale. Obesity should be understood not solely as a condition characterized by surplus adiposity but as a state marked by chronic low-grade inflammation, compromised insulin signaling, and aberrant adipokine secretion, which collectively undermine metabolic flexibility and facilitate the development of type 2 diabetes mellitus, cardiovascular disorders, and cognitive deterioration (1, 2). The process of aging exacerbates these disturbances through the gradual onset of sarcopenia, mitochondrial dysfunction, and neuroinflammaging, thereby impairing both physical performance and cognitive resilience (3). Diabetes, particularly in its diabetes mellitus, exacerbates these consequences by fostering hyperglycemia-induced oxidative stress, endothelial dysfunction, and apoptosis within cardiac and neural tissues (4). Notwithstanding extensive investigations into each of these conditions in isolation, the extant literature seldom integrates them within a systemic framework, thereby revealing a translational deficit in preventive and therapeutic modalities.

Taurine, a semi-essential amino acid that contains sulfur, has attracted scholarly attention due to its diverse functions in maintaining cellular homeostasis, providing antioxidant protection, regulating osmotic balance, and influencing metabolic and cardiovascular pathways (5). Preclinical investigations reveal that taurine enhances insulin sensitivity, promotes the plasticity of adipose tissue, mitigates oxidative stress, and decreases apoptosis within the cardiomyocytes of diabetic and aged rodent models (6, 7). Although clinical evidence remains somewhat sparse, it suggests that taurine supplementation may augment exercise capacity, improve glycemic control, and enhance cardiac function in populations afflicted with metabolic syndrome, diabetes, and heart failure (8, 9). Nonetheless, the majority of studies concentrate on isolated tissues or singular outcomes, often neglecting to address taurine's systemic effects or its potential synergistic interactions with lifestyle interventions, including physical exercise.

Exercise constitutes a robust multi-system intervention, recognized for its capacity to enhance mitochondrial biogenesis, modulate protein kinase B (Akt)/phosphatidylinositol 3-kinase (PI3K) signaling pathways, diminish systemic and neural inflammation, as well as improve both skeletal muscle and cardiovascular functionality (10, 11). Empirical evidence indicates that the synergistic application of aerobic and resistance training can alleviate diabetes-induced myocardial apoptosis, promote neurotrophic factor expression, and enhance glycemic regulation in preclinical models (12). Notwithstanding the shared mechanisms through which taurine and exercise exert their effects especially in the context of oxidative stress regulation, inflammation modulation, and signaling pathway alterations (13, 14), there exists a notable absence of a comprehensive synthesis of research scrutinizing their combined impacts across the domains of obesity, aging, and diabetes, integrating both preclinical and clinical viewpoints.

This review addresses this deficiency by rigorously synthesizing mechanistic insights alongside functional outcomes, elucidating the potential of taurine supplementation and physical exercise as systemic reprogrammers. Significant molecular targets encompass PI3K/Akt signaling pathways, remodeling of adipose tissue, mitochondrial functionality, neuroinflammatory responses, and cardiac apoptotic mechanisms. From a clinical perspective, the synergistic application of these interventions may enhance glycemic regulation, augment both physical and cognitive performance, and foster cardiovascular resilience. By amalgamating evidence from diverse tissues, experimental models, and varying patient cohorts, this review offers a comprehensive framework for comprehending taurine and exercise as complementary modalities, thus providing pragmatic implications for metabolic, cardiovascular, and neuroprotective strategies. The synthesis accentuates the pressing necessity for translational research and rigorously controlled clinical trials to substantiate the multi-systemic benefits and optimize therapeutic protocols.

## 2 Methods (literature search strategy and study selection)

The present review was conducted as a structured narrative synthesis aimed at integrating current mechanistic and clinical evidence regarding the synergistic effects of taurine supplementation and physical exercise in obesity, aging, and diabetes. A comprehensive literature search was performed to identify relevant preclinical and clinical studies examining metabolic, cardiovascular, neurophysiological, and functional outcomes associated with taurine supplementation, exercise interventions, or their combined application.

Electronic databases including PubMed/MEDLINE, Scopus, and Web of Science were systematically searched from database inception through [INSERT MONTH YEAR OF FINAL SEARCH, e.g., January 2026]. Additional studies were identified through manual screening of reference lists from relevant reviews and original research articles to ensure comprehensive coverage of the literature.

Search strategies incorporated combinations of keywords and Medical Subject Headings (MeSH) terms related to the topic, including but not limited to: “taurine,” “exercise,” “physical activity,” “obesity,” “aging,” “senescence,” “diabetes,” “metabolic flexibility,” “adipose tissue,” “mitochondrial function,” “neuroinflammation,” “cardiac remodeling,” “PI3K/Akt signaling,” and “insulin sensitivity.” Boolean operators (AND/OR) were applied to refine search outputs and improve specificity.

Studies were considered eligible if they met the following inclusion criteria: (1) preclinical or clinical investigations evaluating taurine supplementation, structured exercise interventions, or their combination; (2) assessment of metabolic, cardiovascular, neurological, or functional outcomes relevant to obesity, aging, or diabetes; and (3) original research articles published in peer-reviewed journals. Both human and animal studies were included to enable integration of mechanistic insights with translational and clinical findings.

Exclusion criteria included non-English publications, conference abstracts lacking sufficient methodological detail, editorials, opinion papers, and studies not directly addressing taurine-related mechanisms or outcomes relevant to the scope of this review. Studies were selected based on their relevance to key mechanistic pathways emphasized in this manuscript, including adipose tissue plasticity, metabolic adaptability, neuroinflammaging, and cardiac apoptotic signaling.

Given the integrative and conceptual objectives of this review, formal meta-analytic procedures and quantitative synthesis were not performed. Instead, selected studies were qualitatively synthesized to provide a systems-level perspective on the interactions between taurine supplementation and physical exercise across multiple physiological domains.

## 3 Taurine: biology, mechanisms, and therapeutic potential

Taurine (2-aminoethanesulfonic acid) is classified as a sulfur-containing, non-protein amino acid that is prevalent in mammalian tissues. It can be synthesized endogenously and is also obtained from dietary sources, notably from meat and seafood. Taurine is involved in a multitude of physiological processes, including the conjugation of bile acids, osmoregulation, maintenance of calcium homeostasis, stabilization of membranes, provision of antioxidant defense, and modulation of immune responses (15). Its physiological significance becomes particularly apparent in pathological conditions associated with chronic inflammation, metabolic dysregulation, and cellular stress, such as obesity, aging, and diabetes mellitus (5). Within adipose tissue, taurine demonstrates anti-inflammatory and metabolic properties by mitigating pro-inflammatory macrophage infiltration and fostering an M2-like anti-inflammatory phenotype, thereby enhancing insulin sensitivity and metabolic function (16). Additionally, taurine regulates adipogenesis, lipolysis, adipokine secretion, and oxidative stress while modulating transcriptional regulators such as peroxisome proliferator-activated receptor γ (PPARγ) and Peroxisome proliferator-activated receptor gamma coactivator 1-alpha (PGC-1α) to optimize mitochondrial function and energy metabolism (17).

In the context of metabolic disorders, prolonged taurine supplementation has been demonstrated to enhance lipid profiles, diminish fasting insulin and glycated hemoglobin (HbA1c) levels, and improve insulin sensitivity among overweight and obese individuals, particularly when administered at daily dosages approximating 3 g (18). Furthermore, taurine has been evidenced to reduce inflammatory biomarkers, such as C-reactive protein (CRP) and tumor necrosis factor alpha, while simultaneously enhancing fasting glucose and triglyceride levels, thereby indicating significant cardiometabolic advantages (19). Additionally, taurine plays a pivotal role in supporting skeletal muscle physiology and the biological aging process. In various animal models, taurine has been observed to facilitate muscle regeneration, maintain fiber integrity, and mitigate inflammation and oxidative stress, thereby suggesting its potential utility in combating sarcopenia (20). Notwithstanding the encouraging mechanistic and preclinical findings, rigorously controlled clinical trials are imperative to ascertain optimal dosing regimens, tissue-specific effects, and long-term safety across heterogeneous populations.

## 4 Combined effects of taurine supplementation and exercise in overweight individuals

Recent investigations indicate that the incorporation of taurine supplementation alongside meticulously designed exercise regimens may yield synergistic advantages pertaining to critical factors influencing metabolic health in individuals characterized by overweight status, encompassing augmented adipose tissue adaptability, heightened metabolic flexibility, and beneficial preclinical as well as clinical results.

### 4.1 Adipose tissue plasticity

Adipose tissue plasticity pertains to the inherent ability of adipose depots to engage in both functional and morphological adaptations in response to various environmental and metabolic stimuli. In states of obesity and excess weight, adipose tissue experiences dysregulation, manifesting through hypertrophic white adipocytes, heightened pro-inflammatory signaling, and compromised thermogenic capacity. Research has demonstrated that taurine can modulate adipose biology via anti-inflammatory and antioxidant pathways and may affect gene expression associated with adipocyte phenotype and energy metabolism. In studies utilizing rodent models, Guo et al. (21) documented that the administration of taurine facilitates the “browning” of white adipose tissue and enhances the expression of thermogenic markers such as PGC-1α and UCP1, thereby promoting energy expenditure and reducing adiposity. These outcomes are linked to the taurine-induced phosphorylation of AMPK and the activation of mitochondrial biogenesis pathways within adipose tissue. Clinical research substantiates that the integration of taurine supplementation with physical exercise yields significant advantages concerning adipose tissue remodeling in individuals characterized by obesity or overweight. De Carvalho et al. (22) elucidated that an 8-week intervention involving obese females illustrated that the combination of taurine and exercise elevated the levels of anti-inflammatory cytokines like interleukin-10 (IL-10) and IL-15, diminished the expression of pro-inflammatory genes within subcutaneous white adipose tissue (scWAT), and resulted in a reduction of adipocyte size, thereby indicating an enhancement in adipose tissue plasticity that surpasses the effects of exercise alone. Furthermore, taurine-exercise protocols were found to upregulate genes implicated in mitochondrial functionality, lipid oxidation, thereby suggesting an augmentation of metabolic processes within adipocytes.

### 4.2 Metabolic flexibility

Metabolic flexibility, defined as the capacity to alternate between carbohydrate and lipid oxidation in accordance with energy requirements, is frequently diminished in individuals with excess body weight, which exacerbates insulin resistance and ectopic fat accumulation. Taurine has been associated with the enhancement of metabolic flexibility through a variety of mechanisms. Zhao et al. (23) demonstrated that taurine enhances mitochondrial functionality, promotes β-oxidation, and modulates critical regulators of substrate utilization, thereby facilitating improvements in insulin sensitivity and lipid metabolism within skeletal muscle and adipose tissue. Preclinical investigations indicate that these effects may be mediated through the upregulation of insulin signaling components such as insulin receptor substrate-1 and glucose transporter 4. Exercise independently fosters metabolic flexibility by stimulating mitochondrial biogenesis and glucose uptake; however, the concomitant administration of taurine may further enhance these physiological adaptations. Carvalho et al. (24) reported that taurine supplementation elevates post-exercise lipid oxidation and reduces the respiratory quotient in healthy subjects, signifying a transition toward increased fatty acid utilization. In overweight and obese populations, long-term taurine intake (e.g., 3 g/day) has been associated with improvements in glycemic control, insulin sensitivity, and lipid profiles (18), supporting enhanced systemic metabolic flexibility.

### 4.3 Preclinical and clinical evidence

A 8-week investigation was conducted to examine the ramifications of taurine supplementation, either in conjunction with or independent of exercise, on inflammatory and oxidative stress markers present in plasma and scWAT of 16 obese female participants. The subjects were systematically randomized into two distinct cohorts: one receiving taurine exclusively (Tau, *n* = 8) and the other receiving taurine in combination with an exercise regimen (Taurine + Exercise, *n* = 8). Both cohorts were administered a daily dosage of 3 g of taurine, while the Taurine + Exercise group additionally engaged in a structured exercise training program. Various anthropometric measurements, body fat composition, and markers of inflammation and oxidative stress were meticulously evaluated in plasma and scWAT both prior to and following the intervention. The supplementation of taurine resulted in elevated plasma taurine concentrations but exhibited no significant modifications in anthropometric parameters. The Tau group alone demonstrated a reduction in plasma interleukin-6 (IL-6), whereas the Taurine + Exercise group exhibited an increase in anti-inflammatory cytokines IL-10 and IL-15 along with a decrease in IL-1β gene expression in scWAT. Both intervention strategies contributed to a reduction in adipocyte size and an enhancement of connective tissue and multilocular lipid droplets within the scWAT (22) (Table 1). Another study examined the impact of taurine supplementation, physical exercise, or the synergistic combination of both on body composition, mitochondrial functionality, and gene expression within scWAT of 24 obese females (BMI 33.1 ± 2.9 kg/m^2^, age 32.9 ± 6.3 years). Participants were allocated to one of three conditions: taurine only (Tau, *n* = 8), exercise only (Ex, *n* = 8), or a combination of taurine and exercise (TauEx, *n* = 8) for a duration of 8 weeks, during which they received either 3 g of taurine or a placebo daily and engaged in structured exercise when applicable. Measurements of anthropometry, body fat percentage, indirect calorimetry, scWAT mitochondrial respiration, and gene expression were conducted both prior to and following the intervention. No significant changes in anthropometric measurements were detected. The Ex and TauEx groups demonstrated an increase in lipid oxidation coupled with a reduction in the respiratory quotient, with both groups exhibiting an enhancement in scWAT mitochondrial respiratory capacity. Specifically, the TauEx condition led to the upregulation of genes associated with mitochondrial function and adipose tissue browning (CIDEA, PGC1α, PRDM16, UCP1, UCP2) as well as genes implicated in fatty acid oxidation (CPT1, PPARα/γ, LPL, HSL, ACO1/2, ACOX1, CD36) (17).

**Table 1:** Effects of taurine supplementation combined with exercise on metabolic, inflammatory, and functional outcomes in obese humans and animal models.

**Population and design**	**Intervention**	**Results**	**Study**
16 obese women; randomized into Tau (*n* = 8) and Taurine + Exercise (*n* = 8)	Taurine 3 g/day ± exercise, 8 weeks	↑ IL-10 & IL-15 (Taurine + Exercise), ↓ IL-6 (Tau), ↓ IL-1β (Taurine + Exercise); adipocyte size ↓; connective tissue ↑; multilocular droplets ↑	(22)
24 obese women; randomized into Tau (*n* = 8), Exercise (*n* = 8), Taurine + Exercise (*n* = 8)	Taurine 3 g/day ± exercise, 8 weeks	Lipid oxidation ↑ (TauEx & Ex); mitochondrial/browning genes ↑ (TauEx); mitochondrial respiratory capacity ↑; browning gene expression ↑ (TauEx & Ex)	(17)
22 obese women (BMI 32.4 ± 2.0 kg/m^2^, 36.6 ± 6.4 y); double-blind, randomized into GC (*n* = 14) and GTAU (*n* = 8)	Deep Water Running 3 × /week, 50 min, 70–85% HRmax + Taurine 3 g/day	RMR ↑ (DWR); Irisin ↑ 1 h post-exercise (GTAU); Body composition ↔	(25)
11 obese women (BMI 33.12 ± 3.23 kg/m^2^, age 25–45 y); quasi-experimental pre-post	Taurine 50 mg/kg/day for 21 days	Plasma TG ↓ 26%; RER ↓; Fat oxidation ↑; VO_2_max ↑; Time to exhaustion ↑; AT ↔; Body composition ↔	**(** **26** **)**
30 male Wistar rats; obese and lean controls; randomized	Treadmill exercise 20 m/min, 60 min, 5 × /week ± Taurine 2% in drinking water, 7 weeks	Endothelium-dependent relaxation ↑ (exercise & Tau); NOx ↑; TBARS ↓; Cu/Zn SOD & EC SOD ↑; Exercise + Tau ↔ additive effect	**(** **27** **)**
40 T2D women, 53 ± 5 y; randomized into TRX+placebo (TP), TRX+Taurine (TT), Taurine only (T), Control (C)	TRX suspension training 8 weeks + Taurine 3 g/day	Body mass ↓, BMI ↓ (all interventions); BFP ↓↑ (TT > others); FBS ↓ (TP & TT); Insulin ↓ (all interventions); HOMA-IR ↓ (TT > others); HbA1c ↓ (TT & TP); TG ↓ (all); LDL ↓ (all); HDL ↑ (TT only); TC ↓ (TT & TP)	(28)
7–8 healthy men (22–28 y); acute and 7-day supplementation; crossover design	Taurine 1.66 g acutely or 5 g/day for 7 days + 2 h cycling at 60% VO_2_peak	Plasma taurine ↑ 13-fold (acute); Muscle taurine content ↔; Carbohydrate & fat oxidation ↔; Muscle amino acids altered after exercise ↑/↓	(29)
50 male Wistar rats; 40 diabetic (STZ) + 10 healthy controls; randomized	Resistance ladder + treadmill 8 weeks 5 × /week + Taurine 1% in water	LXR ↓ (Tau & Ex+Tau); Total cholesterol ↓ (Ex+Tau); Body weight ↓ (all interventions); BMI ↓ (all interventions)	(30)
Neonate male Swiss MSG-obese mice; randomized into MSG, MSG+Tau, MSG+PE, MSG+PE+Tau	Taurine in drinking water + Swimming training from day 30–90	Triglycerides ↓ 38%; Glucose intolerance ↓~30%; KITT ↑ 79%; Fat accumulation ↔; Insulin resistance ↓ (greatest in PE+Tau)	(31)

↑ Increased, ↓ Decreased, and ↔ No change. Tau, Taurine supplementation; Ex, Exercise; Tau+Ex, Taurine supplementation combined with exercise; TRX, TRX suspension training; DWR, Deep Water Running; PE, Physical exercise; MSG, Monosodium glutamate-induced obese mice; RMR, Resting metabolic rate; VO_2_max, Maximal oxygen consumption; AT, Anaerobic threshold; TG, Triglycerides; HDL, High-density lipoprotein; LDL, Low-density lipoprotein; TC, Total cholesterol; KITT, Insulin sensitivity index; NOx, Nitric oxide metabolites; TBARS, Thiobarbituric acid reactive substances; SOD, Superoxide dismutase; scWAT, Subcutaneous white adipose tissue.

Batitucci et al. (25) assessed the impact of taurine supplementation in conjunction with high-intensity exercise on plasma irisin concentrations in a cohort of 22 sedentary obese females (BMI 32.4 ± 2.0 kg/m^2^, age 36.6 ± 6.4 years). Subjects were allocated randomly to either a control group (GC, *n* = 14) engaging in Deep Water Running (DWR) with a placebo (3 g starch) or a taurine group (GTAU, *n* = 8) participating in DWR with taurine supplementation (3 g) three times per week over 8 weeks, with each session lasting 50 min at an intensity of 70–85% of maximum heart rate. Resting metabolic rate (RMR) was evaluated utilizing indirect calorimetry, body composition was assessed through deuterium oxide, plasma taurine levels were quantified via HPLC, and irisin levels were measured using a Multiplex Kit. No significant alterations were detected in body composition. DWR led to an elevation in RMR irrespective of supplementation, whereas plasma irisin concentrations exhibited a significant increase 1-h post-exercise solely in the taurine-supplemented group (GTAU). In addition, Hasan Abadi et al. (26) evaluated the impact of a 21-day regimen of taurine supplementation (50 mg/kg/day) on fat oxidation, plasma triglyceride levels, and exercise performance in a sample of 11 obese females (mean BMI 33.12 kg/m^2^, age 34.53 ± 6.32 years). The participants underwent pre- and post-assessments utilizing an incremental cycle ergometer protocol, with respiratory gases measured on a breath-by-breath basis to evaluate fat oxidation and anaerobic threshold (AT). Taurine supplementation was found to significantly decrease resting plasma triglycerides by 26% (*P* < 0.01) and to lower the respiratory exchange ratio (RER) during exercise (*P* < 0.05), thereby indicating an enhancement in fat utilization. The proportion of energy derived from fat during exercise exhibited a significant increase, while VO_2_max improved from 16.20 to 19.20 mL/kg/min (*P* < 0.05) and the duration until exhaustion extended from 531 to 573 seconds (*P* < 0.05). No statistically significant alterations were noted in AT, maximum heart rate, or oxygen consumption at AT; however, maximal functional capacity and fat oxidation were augmented following taurine supplementation.

Also, de Moraes et al. (27) demonstrated the impacts of taurine supplementation (2%) and treadmill exercise (20 m/min, 60 min, 5 days/week) on endothelium-dependent vasorelaxation and oxidative stress in obese male Wistar rats. A total of thirty rats were methodically allocated into five distinct groups: lean control, obese sedentary, obese sedentary with taurine supplementation, obese subjected to exercise, and obese subjected to exercise with taurine supplementation. Following a regimen of 7 weeks of intervention subsequent to a 4-week cafeteria diet, the obese sedentary cohort demonstrated significantly impaired endothelium-dependent relaxation (~26%), heightened plasma TBARS levels (138%), diminished NOx concentrations (62%), and a notable reduction in the expression of aortic Cu/Zn SOD and EC SOD proteins (52% and 28%, respectively) in comparison to lean control subjects. Both taurine supplementation and physical exercise independently reinstated endothelial function, augmented NOx levels (by 88% and 108%, respectively), reduced TBARS concentrations (by 18% and 46%), and improved the expression of aortic Cu/Zn and EC SOD proteins. The combination of taurine supplementation and exercise did not yield synergistic effects, suggesting that each intervention individually proficiently alleviates oxidative stress and endothelial dysfunction associated with obesity. A 8-week investigation assessed the impact of TRX suspension training in conjunction with taurine supplementation on body composition, glycemic regulation, and lipid parameters among 40 middle-aged women diagnosed with diabetes mellitus (mean age 53 ± 5 years, average body mass 84.3 ± 5.1 kg). Participants were systematically randomized into four distinct groups: TRX + placebo (TP, *n* = 10), TRX + taurine (TT, *n* = 10), taurine alone (T, *n* = 10), and a control group (C, *n* = 10). All interventions resulted in reductions in body mass, body mass index (BMI), and body fat percentage (BFP), with the TT group exhibiting the most significant decrease in BFP. Fasting blood glucose levels diminished in both the TP and TT groups, while insulin concentrations decreased across all experimental groups. The Homeostasis Model Assessment of Insulin Resistance (HOMA-IR) demonstrated the most substantial improvement within the TT group. Triglycerides (TG), HbA1c, and low-density lipoprotein (LDL) levels were reduced across all interventions, whereas high-density lipoprotein (HDL) levels increased solely in the TT group. Total cholesterol levels experienced a decline in both the TP and TT groups (28).

Galloway et al. (29) indicated the plasma and muscular responses elicited by acute and short-term taurine supplementation during physical exertion. In the initial segment, seven participants (28 ± 3 years, 88 ± 6.6 kg) consumed 1.66 g doses of oral taurine at breakfast and lunch, resulting in an elevation of plasma taurine concentrations from 64 ± 4 μM to maxima of 778 ±1 39 μM by 10 a.m. and 973 ± 181 μM by 1 p.m. In the subsequent phase, eight male subjects (22 ± 1 years, VO_2_peak 4.21 ± 0.16 L/min) engaged in a 2-h cycling session at approximately 60% of their VO_2_peak following a week of either placebo or taurine supplementation (5 g/day). The administration of taurine did not exert a significant impact on resting or post-exercise skeletal muscle taurine levels, glycogen content, or substrate oxidation rates. Nevertheless, taurine supplementation modulated the muscular amino acid responses to physical exercise, influencing the levels of valine, isoleucine, leucine, cystine, glutamate, alanine, and arginine. Furthermore, Ghouroghchi and Orange (30) indicated the impact of taurine supplementation and a synergistic resistance-endurance training regimen on the expression of Liver X Receptor (LXR) and total cholesterol levels in a cohort of 50 male Wistar rats (6 weeks of age, weighing 200–220 g), which included 40 rats with diabetes induced by streptozotocin. The subjects were randomly allocated into four distinct groups: combined exercise (*n* = 10), taurine supplementation (1% in drinking water, *n* = 10), combined exercise plus taurine (*n* = 10), and a diabetic control group (*n* = 10), along with 10 healthy rats serving as controls. The training regimen encompassed ladder climbing and treadmill running at 75% of VO_2_max, conducted five times per week over a period of 8 weeks. The interventions of taurine and exercise were implemented concurrently where appropriate. Following the 8-week intervention period, LXR expression exhibited a statistically significant decrease in the taurine and combined groups in comparison to the control group, with the combined group demonstrating a more pronounced reduction than that observed in the exercise-only group. Furthermore, total cholesterol, body weight, and body mass index (BMI) displayed significant reductions in both the taurine and combined exercise groups relative to the control group, thereby indicating enhanced metabolic markers in the diabetic rats.

**Figure 1:**
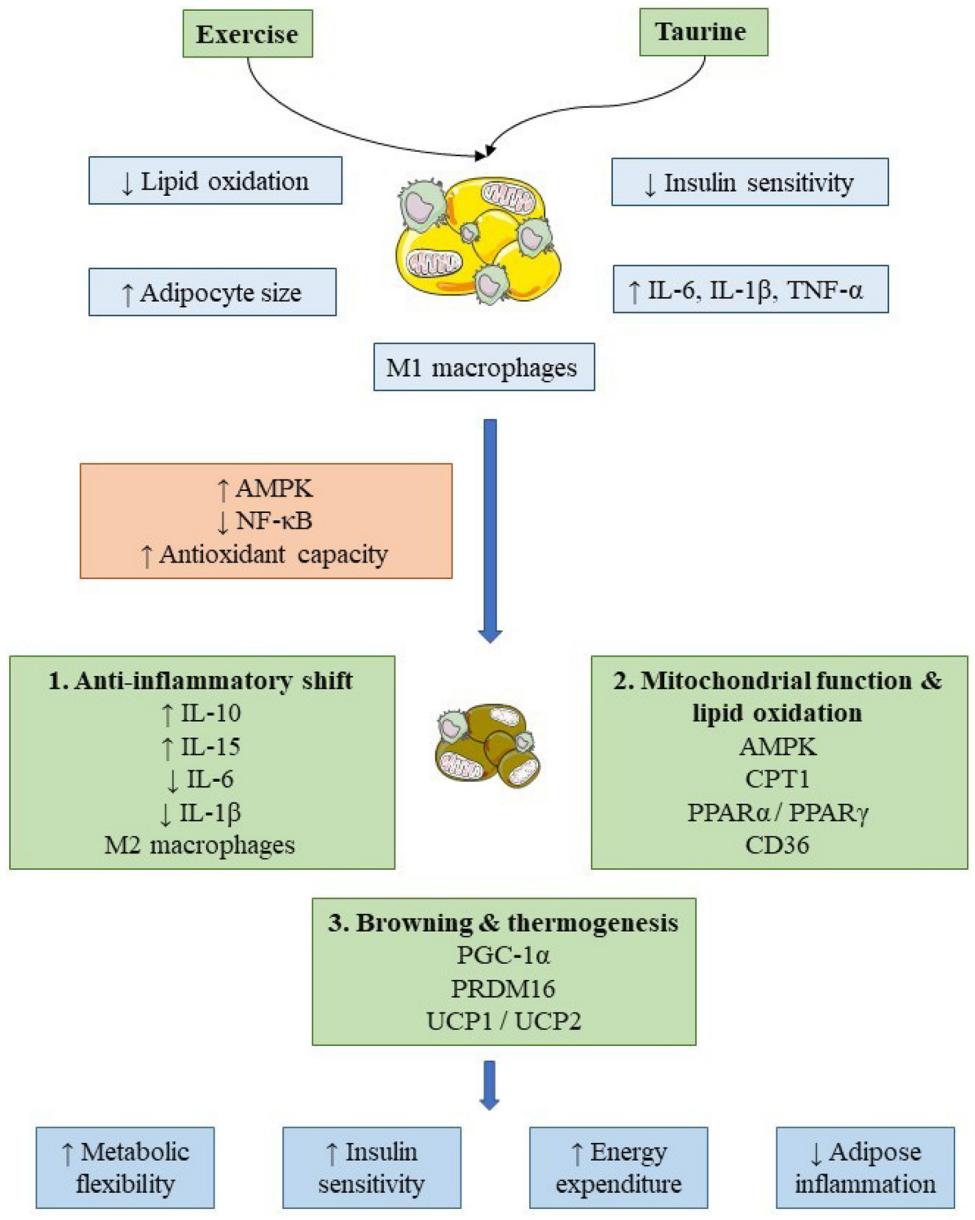
Proposed mechanisms by which taurine supplementation and physical exercise influence adipose tissue plasticity and metabolic flexibility. Schematic representation illustrating proposed additive and potentially complementary effects of taurine supplementation and physical exercise on adipose tissue remodeling in overweight and obese states. In the pathological condition, adipose tissue is characterized by adipocyte hypertrophy, reduced mitochondrial content (↓ mitochondrial biogenesis), increased immune cell infiltration (↑ pro-inflammatory macrophages), and elevated pro-inflammatory cytokine signaling (↑ NF-κB activity), contributing to impaired lipid oxidation and decreased insulin sensitivity. Taurine and exercise are depicted as converging modulators of metabolic and anti-inflammatory pathways, including activation of AMPK (↑) and PGC-1α signaling (↑), alongside suppression of NF-κB–mediated inflammatory responses (↓). These interactions are associated with enhanced mitochondrial biogenesis (↑), increased fatty acid oxidation (↑), promotion of white-to-beige adipocyte phenotypic conversion (↑), and shifts toward anti-inflammatory macrophage polarization (↑ M2-like phenotype; ↓ M1-like phenotype). Solid arrows indicate pathways supported by experimental evidence, whereas dashed arrows represent proposed or emerging interactions inferred primarily from preclinical studies. Upward arrows (↑) denote activation or increased activity/expression, while downward arrows (↓) indicate inhibition or reduction. Collectively, these adaptations are associated with improved metabolic flexibility and adipose tissue function, which may occur independently of substantial body weight reduction.

Another investigation assessed the impact of taurine supplementation and physical exercise, either individually or in conjunction, on obesity and glucose homeostasis in male Swiss mice rendered obese via monosodium glutamate (MSG) administration. During the neonatal phase, the mice were administered either MSG or saline as a control; subsequently, between days 30 and 90, the MSG-treated mice were allocated to receive taurine via drinking water (MSG TAU), engage in exercise training (MSG PE), or partake in both interventions (MSG PE TAU). The mice subjected to MSG treatment exhibited characteristics of obesity, hypertriglyceridemia, glucose intolerance, and insulin resistance. Administration of taurine or participation in exercise independently resulted in a reduction of plasma triglycerides by 38%, an enhancement of glucose tolerance by approximately 30%, and an increase in insulin sensitivity (KITT) by 79% in the MSG-treated cohort, without influencing adipose tissue accumulation. The synergistic intervention of taurine and exercise yielded an even greater decrease in insulin resistance compared to the effects of each intervention alone, thereby suggesting that the concurrent implementation of both strategies confers superior metabolic advantages in the context of obesity in mice (31). Overall, taurine supplementation, whether administered independently or in conjunction with physical exercise, orchestrates a myriad of molecular pathways to ameliorate metabolic health in the contexts of obesity, diabetes, and senescence. Within adipose tissue, taurine facilitates an increase in plasticity by stimulating the browning of white adipose tissue, diminishing the size of adipocytes, and elevating the expression of thermogenic markers such as PGC-1α, UCP1/2, PRDM16, and CIDEA, while also enhancing lipid oxidation through mechanisms involving CPT1, PPARα/γ, LPL, HSL, ACO1/2, and ACOX1. Additionally, taurine mitigates inflammatory responses by reducing levels of IL-6 and IL-1β while simultaneously augmenting the expression of IL-10 and IL-15. In skeletal muscle, taurine exerts its influence on amino acid metabolism and enhances the capacity for mitochondrial respiration, without affecting muscle taurine concentrations or substrate oxidation rates. The cardiovascular implications include enhanced endothelium-dependent relaxation through increased bioavailability of nitric oxide and elevated expression of Cu/Zn superoxide dismutase and extracellular superoxide dismutase. Moreover, taurine plays a significant role in the modulation of glucose homeostasis, insulin sensitivity, lipid metabolism, and liver X receptor expression, while synergistically collaborating with high-intensity and resistance training to amplify fat oxidation, respiratory exchange ratio, maximum oxygen uptake, and overall metabolic adaptability (Figure 1).

Across preclinical and clinical studies, taurine supplementation combined with exercise generally improves inflammatory status, lipid metabolism, and mitochondrial-related outcomes; however, the magnitude and interaction pattern of these effects remain heterogeneous. Several investigations report enhanced anti-inflammatory signaling, increased lipid oxidation, and improved insulin sensitivity with combined interventions, yet others demonstrate comparable benefits from taurine or exercise alone without clear additive effects, particularly in endothelial and oxidative stress outcomes. Functional outcomes such as body composition and exercise performance also vary considerably, with metabolic improvements frequently occurring in the absence of significant anthropometric changes. These inconsistencies appear largely attributable to methodological variability, including species differences between animal and human models, substantial variation in taurine dosing strategies and administration routes, heterogeneity in exercise modality and intensity, and differences in intervention duration and baseline metabolic status of participants. Consequently, it remains unresolved whether taurine and exercise exert synergistic physiological effects or primarily influence overlapping regulatory pathways. Furthermore, mechanistic evidence linking molecular adaptations to clinically meaningful outcomes in humans remains limited, and optimal dosing strategies for enhancing exercise-induced metabolic adaptations have not been established. Collectively, current evidence supports context-dependent rather than uniformly synergistic interactions between taurine supplementation and exercise, highlighting the need for standardized protocols and mechanistically integrated clinical trials.

## 5 Combined effects of taurine supplementation and exercise in older adults

### 5.1 Neuroinflammation and cognitive health

Aging is intricately linked to a persistent, low-grade inflammatory condition within the central nervous system, referred to as neuroinflammaging, which plays a substantial role in cognitive deterioration, synaptic impairment, and an increased susceptibility to neurodegenerative disorders in the elderly population. This phenomenon is characterized by the prolonged activation of microglia and astrocytes, an augmented production of pro-inflammatory cytokines, as well as intensified oxidative stress and mitochondrial dysfunction (3, 32). Taurine, a sulfur-containing β-amino acid that is particularly prevalent in the brain, has garnered increasing scholarly interest due to its neuroprotective, anti-inflammatory, and antioxidant characteristics. Taurine modulates intracellular calcium equilibrium, maintains mitochondrial integrity, and inhibits critical neuroinflammatory pathways (5, 33). Age-associated reductions in taurine levels have been correlated with diminished synaptic plasticity and cognitive impairments, indicating that taurine supplementation may potentially reinstate neuronal resilience and mitigate the effects of neuroinflammaging (34).

Regular engagement in physical exercise constitutes one of the most efficacious non-pharmacological interventions to mitigate neuroinflammaging and to safeguard cognitive integrity throughout the aging process. Both aerobic and resistance training have been shown to diminish microglial activation, reduce levels of pro-inflammatory cytokines, and enhance the expression of neurotrophic factors such as BDNF and Insulin-like Growth Factor 1 (IGF-1), thereby fostering synaptic plasticity and cognitive function in the elderly population (35, 36). Furthermore, exercise contributes to the improvement of cerebral insulin sensitivity and promotes mitochondrial biogenesis, thereby alleviating age-related neuroinflammatory mechanisms. Recent findings suggest a complementary relationship between taurine supplementation and physical exercise in influencing neuroinflammaging and cognitive performance. Taurine may enhance the neuroprotective effects of exercise by reducing oxidative stress through the attenuation of excessive reactive oxygen species (ROS) production, bolstering intrinsic antioxidant defenses, and stabilizing the balance between excitatory and inhibitory neurotransmission via the modulation of glutamatergic and GABAergic signaling pathways (37). In contrast, exercise may improve the bioavailability and transport of taurine into neural tissues by enhancing cerebral blood flow and the activity of amino acid transporters (38).

Investigations indicate that the concurrent administration of taurine supplementation alongside exercise yields a more pronounced attenuation of hippocampal inflammation, diminishes microglial hyperactivation, and enhances cognitive learning and memory capabilities when compared to either intervention in isolation (38, 39). The advantages of this combined strategy seem to be mediated by the coordinated suppression of NF-κB signaling pathways, a reduction in oxidative stress, an augmentation of antioxidant enzyme activities, and the activation of the BDNF-TrkB signaling cascade. In summary, the amalgamation of taurine supplementation with systematic exercise regimens constitutes a promising methodology to alleviate neuroinflammaging and maintain cognitive faculties in the elderly population, especially within the framework of obesity, metabolic impairments, and diabetes. This integrative approach is congruent with the tenets of predictive, preventive, and personalized medicine, as it specifically targets the common molecular pathways that underlie inflammation, oxidative damage, mitochondrial dysfunction, and the decline in neuroplasticity associated with aging.

### 5.2 Preclinical and clinical evidence

Immunosenescence is intricately linked to cognitive deterioration and neurodegenerative processes, which may be alleviated through non-pharmacological anti-inflammatory interventions such as physical exercise and taurine supplementation. Considering the age-related decline in endogenous taurine concentrations, this investigation assessed the impacts of taurine supplementation, either in isolation or in conjunction with exercise, on oxidative stress, extracellular matrix remodeling, immune metrics, neurotrophins, cognitive function, and physical fitness among elderly female participants. A total of 48 women (mean age 83.6 years) were assigned to one of four groups: exercise only, taurine supplementation, a combination of exercise and taurine supplementation, or a control group for a duration of 14 weeks. The exercise regimen was conducted bi-weekly, whereas taurine was administered on a daily basis at a dosage of 1.5 grams. Various biomarkers, including myeloperoxidase, matrix metalloproteinase-9, neurotrophins, and white blood cell counts, were evaluated in conjunction with cognitive performance metrics (MoCA) and measures of physical fitness. Taurine supplementation resulted in a reduction of myeloperoxidase and matrix metalloproteinase-9 levels, while cognitive performance scores exhibited a decline solely within the control group. The combination of exercise and taurine supplementation led to enhancements in agility and aerobic capacity (40) (Table 2). Immunosenescence is correlated with an augmentation in blood–brain barrier (BBB) permeability, which contributes to the deterioration of cognitive functions and the progression of neurodegenerative conditions. Chupel et al. (39) assessed the anti-inflammatory impact of physical exercise and taurine supplementation on peripheral BBB indicators, inflammatory cytokines, and cognitive capabilities among elderly female participants. A total of 48 subjects (mean age 83.6 years) were allocated to groups receiving combined exercise training, taurine supplementation, a combination of exercise and taurine, or a control condition over a period of 14 weeks. The exercise regimen was conducted biweekly, while taurine was administered daily at a dosage of 1.5 grams. The circulating inflammatory cytokines, alongside serum S100β and neuron-specific enolase as markers associated with BBB integrity, were evaluated pre- and post-intervention, and cognitive functioning was assessed via the Mini-Mental State Examination. Engagement in physical exercise resulted in a reduction of pro-inflammatory cytokines and inflammatory ratios, whereas taurine supplementation led to a decrease in the IL-1β/IL-1ra ratio. Enhancements in cognitive performance were observed solely in the group receiving both exercise and taurine, while markers indicative of BBB integrity were preserved across all intervention groups, albeit exhibiting a tendency to deteriorate in the control group.

**Table 2:** Key outcomes of taurine supplementation and exercise in older adults.

**Population and design**	**Intervention**	**Results**	**Study**
48 elderly women (83.6 ± 7.0 years), randomized into 4 groups: Exercise only (EO, *n* = 13), Taurine only (TS, *n* = 12), Exercise + Taurine (ETTS, *n* = 11), Control (CG, *n* = 12); 14-week intervention	EO: Multicomponent exercise twice/week TS: Taurine 1.5 g/day ETTS: Exercise + Taurine	MPO and MMP-9 decreased in TS (*P* < 0.05) WBC unchanged overall; lymphocytes decreased in TS, monocytes increased in CG (*P* < 0.05) MoCA declined in CG (*P* < 0.05) Physical fitness (agility, aerobic capacity) improved in ETTS No changes in BDNF; NGF undetectable	(40)
48 elderly women (83.6 ± 6.9 years), randomized into 4 groups: Combined exercise training (CET, *n* = 13), Taurine (TAU, *n* = 12), Exercise + Taurine (CET+TAU, *n* = 11), Control (CG, *n* = 12); 14-week intervention	CET: Multi-modal exercise twice/week TAU: Taurine 1.5 g/day CET+TAU: Exercise + Taurine	CET reduced TNF-α, IL-6, IL-1β/IL-1ra, IL-6/IL-10, TNF-α/IL-10 ratios (*P* < 0.05) TAU decreased IL-1β/IL-1ra ratio (*P* < 0.05) MMSE improved only in CET+TAU group (*P* < 0.05) S100β maintained in interventions; slight increase in CG NSE increased only in TAU group (*P* < 0.05)	(39)
31 healthy active men (*n* = 16, 56 ± 6 yrs) and women (*n* = 15, 52 ± 7.5 yrs); randomized, double-blind, placebo-controlled crossover design; 28-day supplementation cycles with 1-week washout	Multi-nutrient supplement: branched-chain amino acids, taurine, anti-inflammatory plant extracts, B vitamins	IL-6 decreased in men and women Perceived energy improved in both sexes Men: reductions in alpha-1-antichymotrypsin, creatine kinase, general pain, joint pain; increased vertical jump power and grip strength Women: reduced anxiety and improved balance	(41)
Patients with heart failure, LVEF <50%, NYHA II–III; randomized to taurine (*n* not reported) or placebo; 2-week intervention	Taurine 500 mg orally, 3 × /day; incremental treadmill exercise before and after supplementation	Aurine group: decreased CRP and platelet counts pre- and post-exercise (*P* < 0.05) Reduced atherogenic indices (CRI-I, CRI-II, AC) pre- and post-exercise (*P* < 0.05) Placebo group: inflammatory and atherogenic markers increased or unchanged	(42)
35 sedentary older women (60–75 yrs; BMI 30–40 kg/m^2^; sarcopenia) randomized into 4 groups: placebo (GPLA), taurine (GTAU), placebo + exercise (GPLA+EX), taurine + exercise (GTAU+EX); 16-week intervention	Multicomponent exercise 3 × /week +/– taurine 3 g/day	GPLA+EX: blood glucose decreased (−13.6 mg/dL), insulin decreased (−8.3 μU/mL), improved insulin sensitivity (−2.5) GTAU+EX: insulin decreased (−7.8 μU/mL) Molecular network: decreased monosaccharides (glucopyranose, methyl-hexofuranose, talose) post-intervention	(43)
35 women >60 yrs (BMI 30–40 kg/m^2^; appendicular lean mass <0.512 kg/BMI; handgrip <0.56 kgf), randomized into exercise + taurine (GTAU+EX), exercise + placebo (GPLA+EX), taurine only (GTAU), placebo (GPLA); 16-week intervention	Multicomponent training 3 × /week + taurine 3 g/day	GTAU+EX: adipocyte area decreased (-5598.53 μm^2^, *P* = 0.014) GPLA+EX and GTAU+EX: increased REE (151.4 kcal and 334.9 kcal, respectively) Plasma irisin increased trend (*P* = 0.053)	(44)
43 postmenopausal women randomized: C (*n* = 13), T (*n* = 8), TE (*n* = 13), E (*n* = 9); 8-week intervention	TE: Concurrent training (50 min resistance + 30 min aerobic, 3 × /week) + taurine 1.5 g/day	TE: ↓ total body fat (−4.1%) & trunk fat (−4.4%), ↑ lean body mass (3.9 kg) ↓ total cholesterol (−0.8 mg/dL) & LDL-C (−13.6 mg/dL) compared to C and T	(45)
52 male Wistar rats (15 mo), 4 sedentary & 4 exercise groups; 8-week intervention	Taurine 800 mg/kg/day orally +/– exercise; comparisons with DDD water, 1% Tween 80, vitamin E	Exercise groups: ↓ body weight & plasma glucose Taurine: ↑ TC (sedentary), ↓ TG (sedentary), ↓ BUN (exercise) Exercise: ↑ BUN, ALT, heart ROW	(46)
112 patients (50 surgical, 62 endovascular), age 50–75 yrs; pilot study	Interval Walking Program + Tribulus Terrestris + Taurine 3–5 g + ALA 1,800 mg	83% patients with above-knee bypass fully rehabilitated vs. 46.6% below-knee (*P* < 0.05) Supports improved bypass potency, vascular rehabilitation, and link to lifestyle modification & serum testosterone	(47)

↑ Increased, ↓ Decreased. EO, Exercise Only; TS, Taurine Supplementation; ETTS, Exercise + Taurine Supplementation; CG, Control Group; CET, Combined Exercise Training; TAU, Taurine; CET+TAU, Exercise + Taurine; MMSE, Mini-Mental State Examination; MPO, Myeloperoxidase; MMP-9, Matrix Metalloproteinase-9; BDNF, Brain-Derived Neurotrophic Factor; NGF, Nerve Growth Factor; GPLA, Placebo; GTAU, Taurine; GPLA+EX, Placebo + Exercise; GTAU+EX, Taurine + Exercise; REE, Resting Energy Expenditure; TE, Taurine + Exercise; C, Control; T, Taurine; DXA, Dual-Energy X-ray Absorptiometry; LDL-C, Low-Density Lipoprotein Cholesterol; TC, Total Cholesterol; TG, Triglycerides; ROW, Relative Organ Weight; HF, Heart Failure; CRP, C-Reactive Protein; CRI-I/II, Castelli's Risk Index I/II; AC, Atherogenic Coefficient; ALA, Alpha-Lipoic Acid; TT, Tribulus Terrestris.

Dunn-Lewis et al. (41) demonstrated the impacts of a multi-nutrient supplement comprising branched-chain amino acids, taurine, anti-inflammatory phytochemicals, and B vitamins on inflammation, endothelial functionality, physical performance, and psychological state in middle-aged individuals. Thirty-one healthy, physically active males and females participated in two distinct 28-day supplementation intervals (placebo and active supplement) interspersed with a washout phase. The evaluated outcomes encompassed inflammatory biomarkers, brachial artery flow-mediated dilation, physical performance assessments, balance, and perceptual evaluations. The supplementation regimen significantly diminished circulating IL-6 concentrations in both genders and enhanced perceived energy levels. In males, there were notable reductions in alpha-1-antichymotrypsin, creatine kinase, general pain, and joint pain, in conjunction with enhancements in vertical jump power and handgrip strength. In females, the supplementation resulted in decreased anxiety levels and improved balance. Gender-specific responses were apparent across inflammatory, neuromuscular, and perceptual metrics. Also, Ahmadian et al. (42) examined the anti-inflammatory and anti-atherogenic properties of taurine supplementation administered prior to and following incremental exercise in individuals diagnosed with heart failure. Subjects exhibiting a diminished left ventricular ejection fraction (<50%) and categorized as NYHA functional class II or III were allocated to either taurine or placebo cohorts. The taurine cohort received oral taurine (500 mg, thrice daily) over a 2-week duration, while the placebo cohort was administered starch supplementation adhering to the same regimen. Participants engaged in incremental treadmill exercise assessments both pre- and post-supplementation utilizing a modified Bruce protocol. Inflammatory biomarkers, encompassing CRP and platelet counts, in addition to atherogenic indices such as Castelli's Risk Index I and II and the atherogenic coefficient, were evaluated. Taurine supplementation demonstrably diminished inflammatory and atherogenic indices both at rest and subsequent to exercise, whereas these parameters either escalated or remained static within the placebo cohort.

Abud et al. (43) investigated the impact of physical exercise, with or without the inclusion of taurine supplementation, on metabolic outcomes in elderly women diagnosed with sarcopenic obesity. A total of 35 sedentary women, aged between 60 and 75 years, exhibiting both obesity and sarcopenia, were systematically assigned to either placebo, taurine, placebo combined with exercise, or taurine combined with exercise cohorts. Over a duration of 16 weeks, participants engaged in multicomponent exercise training three times weekly and/or received taurine supplementation at a dosage of 3 g/day. Measured outcomes encompassed blood glucose levels, insulin concentrations, insulin sensitivity, and untargeted metabolomic profiles. The exercise intervention in conjunction with placebo significantly diminished blood glucose and insulin levels while enhancing insulin sensitivity; conversely, the exercise combined with taurine also resulted in a reduction of insulin concentrations. Metabolomic analyses indicated a post-intervention chemical homogeneity across the groups, with molecular networking suggesting a decrease in circulating monosaccharides subsequent to the exercise regimen. In addition, Ortiz et al. (44) examined the implications of physical exercise in conjunction with taurine supplementation on the morphological characteristics of adipocytes in older females diagnosed with sarcopenic obesity. Thirty-five women aged over 60, characterized by obesity and sarcopenia, were assigned to one of four groups: exercise combined with taurine, exercise paired with placebo, taurine administered alone, or a placebo group, all participating in a 16-week intervention. The intervention protocol encompassed multicomponent training conducted three times weekly, alongside taurine supplementation at a dosage of 3 g/day. Prior to and subsequent to the intervention, subcutaneous adipose tissue biopsies, measurements of resting energy expenditure, plasma irisin concentrations, and comprehensive metabolic evaluations were executed. Initial analyses revealed a statistically significant decrease in adipocyte area exclusively within the taurine and exercise cohort. Both groups engaging in exercise exhibited enhancements in resting energy expenditure, with a more pronounced effect noted when exercise was supplemented with taurine. These physiological alterations were correlated with a tendency toward elevated levels of circulating irisin.

Also, Buonani et al. (45) documented the impact of concurrent training, in conjunction with taurine supplementation, on body composition and lipid metabolism among postmenopausal women. A total of 43 participants were systematically allocated to either a control group, a taurine-only group, an exercise plus placebo group, or an exercise plus taurine group over a duration of 8 weeks. Taurine supplementation was administered at a dosage of 1.5 g/day, while the concurrent training regimen encompassed both resistance and aerobic exercises conducted three times per week. Body composition was evaluated utilizing dual-energy X-ray absorptiometry (DXA), and lipid profiles were meticulously analyzed. The exercise plus taurine cohort demonstrated statistically significant reductions in both total and trunk body fat percentages, as well as enhanced increases in lean body mass, in comparison to the control and taurine-only groups. Furthermore, this cohort exhibited a more pronounced decrease in total cholesterol levels and a significant reduction in low-density lipoprotein cholesterol when contrasted with the control and taurine-only groups. Furthermore, Onsri and Srisawat (46) evaluated the impact of taurine supplementation in conjunction with physical exercise on body mass, organ weights, and biochemical indicators in aged male rats. Fifteen-month-old Wistar rats were systematically allocated to either sedentary or exercise conditions and underwent daily oral administration of water, vehicle, vitamin E, or taurine (800 mg/kg) over a duration of 8 weeks. Body mass was monitored on a weekly basis, and plasma metabolic, renal, and hepatic markers were analyzed upon the study's conclusion, in addition to the relative weights of various organs. Physical exercise resulted in a reduction of final body mass and plasma glucose concentrations in comparison to sedentary conditions. Taurine supplementation diminished plasma triglyceride levels in sedentary rats and lowered blood urea nitrogen concentrations in exercised rats, thereby suggesting enhancements in metabolic and renal profiles. Exercise led to an increase in certain relative organ weights and liver-associated enzymes, while taurine selectively influenced lipid and renal markers.

**Figure 2:**
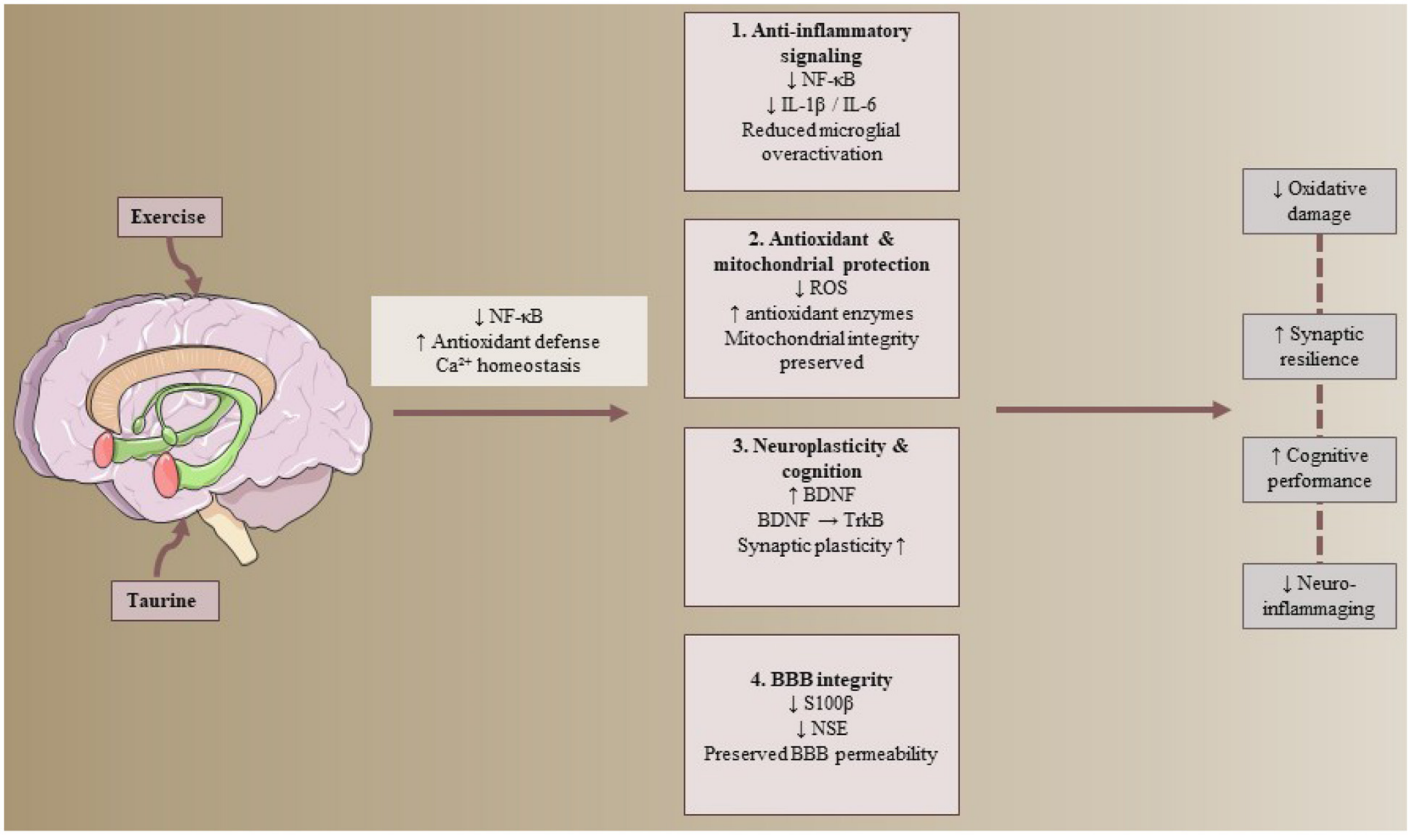
Proposed interactions between taurine supplementation and physical exercise in modulating neuroinflammaging and neuroprotection during aging. Schematic illustration depicting potential additive or complementary effects of taurine supplementation and physical exercise on age-associated neuroinflammation and cognitive decline within the central nervous system. Aging-related pathological features include chronic microglial activation (↑), increased oxidative stress (↑ reactive oxygen species), elevated pro-inflammatory cytokine production (↑), disruption of blood–brain barrier integrity (↓), and reduced synaptic plasticity and neuronal resilience (↓). Taurine and exercise are presented as converging modulators that influence key signaling pathways, including suppression of NF-κB-mediated inflammatory signaling (↓), reduction of oxidative damage (↓), and stabilization of intracellular calcium homeostasis and mitochondrial function (↑ mitochondrial efficiency). These processes are associated with enhanced neurotrophin signaling, particularly activation of BDNF-TrkB pathways (↑), contributing to improved synaptic integrity (↑) and neuronal adaptability (↑). Solid arrows represent pathways supported by experimental findings, whereas dashed arrows indicate proposed or emerging mechanisms derived primarily from preclinical models. Upward arrows (↑) denote increased activity, expression, or functional enhancement, while downward arrows (↓) indicate inhibition or reduction. Collectively, these mechanisms are associated with potential neuroprotective effects and preservation of cognitive function; however, the degree of interaction between taurine and exercise remains incompletely defined, and evidence derives largely from animal studies with limited direct human confirmation.

An investigation assessed the impacts of an Interval Walking Training Program supplemented with Tribulus Terrestris, taurine (3–5 g), and a high dosage of alpha-lipoic acid (ALA, 1,800 mg) on vascular functionality and rehabilitation outcomes in individuals post-surgical or endovascular interventions. A total of 112 participants, aged between 50 and 75 years, were included in the study: 50 underwent surgical bypass procedures (aortic-iliac, aortic-femoral, femoral distal vein) while 62 received endovascular treatment for the iliac region. The participants were administered supplementation concomitant with a walking-based rehabilitation regimen (30–45 min per session). Findings indicated that 83% of patients who underwent above-knee bypass attained full rehabilitation, in contrast to 46.6% of those who underwent below-knee bypass (*P* < 0.05). A history of hypertension, hyperlipidemia, smoking, and diabetes was prevalent among the participants; however, no statistically significant differences were observed between the groups (47). Totally, taurine supplementation, whether administered singularly or in conjunction with physical activity, manifests diverse molecular impacts on the domains of aging, metabolic processes, and cardiovascular health. Taurine influences oxidative stress by diminishing reactive oxygen species and augmenting the activity of antioxidant enzymes, concurrently mitigating chronic inflammation via the downregulation of NF-κB signaling pathways and pro-inflammatory cytokines such as IL-1β, IL-6, and TNF-α. In adipose tissue, taurine and physical exercise act in concert to diminish adipocyte hypertrophy, enhance resting energy expenditure, and elevate levels of irisin, which collectively foster improved metabolic flexibility and lipolysis. Within skeletal muscle and the context of sarcopenic obesity, taurine improves insulin sensitivity and regulates glucose homeostasis, in part through the modulation of monosaccharide metabolism. Cardiovascular advantages encompass diminished atherogenic markers and enhanced endothelial function, presumably mediated by anti-inflammatory and antioxidative processes. In the realm of the central nervous system, taurine lessens the effects of neuroinflammaging and sustains the integrity of the blood–brain barrier, thereby supporting cognitive functions. Collectively, these investigations underscore taurine's capacity to amplify exercise-induced molecular adaptations across metabolic, cardiovascular, and neuroprotective pathways in the context of aging and disease (Figure 2).

Evidence from studies in older populations indicates that taurine supplementation and exercise produce broadly consistent anti-inflammatory and functional benefits, yet the pattern of interaction between the two interventions varies across physiological domains. Human trials in elderly women consistently demonstrate that exercise is the primary driver of systemic anti-inflammatory effects, while taurine supplementation appears to exert more selective modulation of immune and oxidative stress markers. For example, reductions in pro-inflammatory cytokines are most pronounced with exercise-based interventions, whereas taurine alone preferentially influences specific inflammatory ratios or oxidative stress markers without uniformly improving functional outcomes. In contrast, improvements in cognitive performance and physical fitness tend to emerge primarily when taurine is combined with exercise, suggesting complementary rather than independent effects on neurofunctional outcomes.

Metabolic and body composition outcomes exhibit greater variability across studies. In populations with sarcopenic obesity or postmenopausal status, combined interventions frequently produce greater reductions in adipocyte size, fat mass, and lipid-related risk markers than either intervention alone; however, some investigations report metabolic improvements driven largely by exercise with taurine providing limited additional benefit.

Similarly, while taurine supplementation has been associated with reductions in inflammatory or atherogenic indices in cardiovascular populations, multi-nutrient supplementation studies complicate interpretation of taurine-specific effects due to co-administered bioactive compounds.

Several methodological factors account for these divergent findings. Intervention duration ranges from short-term supplementation to multi-month training programs, exercise prescriptions differ substantially in modality and frequency, and taurine dosing varies across studies despite similar age ranges. Additionally, participant health status differs markedly across cohorts, spanning healthy active older adults, frail elderly women, heart failure patients, and individuals with sarcopenic obesity, thereby influencing responsiveness to intervention.

Collectively, the evidence suggests that in older adults, exercise serves as the dominant stimulus for systemic metabolic and inflammatory adaptation, while taurine supplementation may enhance specific tissue-level, cognitive, or body composition responses under certain physiological conditions. However, it remains unresolved whether these benefits reflect true synergistic mechanisms or context-dependent amplification of exercise-induced adaptations, and mechanistic human data linking molecular changes to functional aging outcomes remain limited.

## 6 Combined effects of taurine supplementation and exercise in individuals with diabetes

### 6.1 Cardiac remodeling and PI3K/Akt pathway

Diabetic cardiomyopathy is characterized by both structural and molecular modifications within the myocardium, which encompass heightened cardiomyocyte apoptosis and maladaptive remodeling. A pivotal survival pathway that plays a crucial role in mitigating this detrimental process is the phosphoinositide 3-kinase (PI3K)/Akt signaling pathway (12), which facilitates cardiomyocyte viability and impedes pro-apoptotic signals such as caspase-3 and caspase-9. In the context of diabetes mellitus, augmented levels of glucose and lipid toxicity inhibit the activation of the PI3K/Akt pathway, resulting in diminished phosphorylation of Akt and an escalation in apoptosis, thereby exacerbating the deterioration of cardiac architecture and functionality. Both Sedaghat et al. (12) and Wang et al. (48) elucidated in diabetic rat models that the concomitant administration of taurine alongside aerobic and resistance exercise significantly augments Akt expression while simultaneously attenuating markers of apoptosis when juxtaposed with diabetic control groups. The synergistic intervention reinstated Akt signaling with greater efficacy than either modality employed in isolation, thereby alleviating cardiomyocyte apoptosis and positively influencing cardiac remodeling through enhanced pro-survival signaling and diminished caspase activity (12, 48). Concurrently, it has been established that exercise alone activates IGF-I receptor-mediated PI3K/Akt pathways and enhances the expression of survival proteins such as Bcl-2, thereby providing further protection against cardiac damage induced by diabetes (49). These observations imply that taurine and exercise function in a complementary manner to restore compromised signaling, inhibit apoptosis, and facilitate adaptive cardiac remodeling in the context of diabetes.

### 6.2 mTOR pathway and metabolic regulation

The mechanistic target of rapamycin (mTOR) is a highly conserved serine/threonine kinase that serves as a central regulator of cellular metabolism, growth, and energy sensing (50). Functionally, mTOR operates within two distinct multiprotein complexes: mTOR complex 1 (mTORC1), which primarily controls anabolic metabolism and nutrient-responsive signaling, and mTOR complex 2 (mTORC2), which regulates cytoskeletal organization, insulin signaling, and cellular survival pathways. Through these complexes, mTOR integrates signals derived from nutrient availability, growth factors, energy status, and mechanical stimuli to coordinate metabolic adaptation at both cellular and systemic levels (50).

mTORC1 is particularly important for metabolic regulation because it promotes protein synthesis, lipid biosynthesis, and mitochondrial function while inhibiting autophagy (51). Activation of mTORC1 occurs in response to amino acids, insulin-mediated PI3K/Akt signaling, and sufficient cellular energy availability, thereby supporting anabolic processes when resources are abundant. Conversely, suppression of mTORC1 activity during nutrient deprivation or energetic stress promotes catabolic pathways such as autophagy, allowing cells to recycle substrates and maintain energy balance. This dynamic regulation enables metabolic flexibility, ensuring that cells adjust fuel utilization and biosynthetic activity according to environmental conditions (51).

In metabolic tissues, mTOR signaling influences glucose homeostasis, adipose tissue remodeling, and skeletal muscle adaptation (52). For example, mTORC1 activation enhances glycolysis and lipid storage pathways, contributing to adipocyte hypertrophy under chronic nutrient excess. However, excessive or sustained mTORC1 activation has been associated with insulin resistance and metabolic dysfunction, partly through feedback inhibition of insulin signaling. In skeletal muscle, mTOR activation promotes protein synthesis and mitochondrial adaptations that support increased metabolic capacity, highlighting the context-dependent nature of mTOR signaling (52, 53).

Physical exercise represents a key physiological regulator of mTOR activity (54). Resistance exercise transiently activates mTORC1, stimulating muscle protein synthesis and remodeling, whereas endurance exercise can modulate mTOR signaling through energy-sensing pathways such as AMPK, promoting mitochondrial biogenesis and improved oxidative metabolism. The balance between mTOR activation and inhibition is therefore essential for optimal metabolic adaptation to training stimuli (54).

Emerging evidence suggests that taurine may also influence metabolic regulation through modulation of pathways converging on mTOR signaling (55, 56). Taurine has been associated with improvements in mitochondrial function, oxidative stress regulation, and calcium homeostasis, factors that indirectly impact mTOR activity and downstream metabolic processes. Although mechanistic interactions between taurine, exercise, and mTOR remain incompletely characterized, current data suggest that coordinated modulation of mTOR-dependent anabolic and catabolic signaling may contribute to improved metabolic flexibility and tissue-specific adaptations (56, 57).

### 6.3 Preclinical and clinical evidence on PI3K/Akt pathway

An investigation examined the ramifications of taurine supplementation when integrated with aerobic and resistance exercise (CARE) on myocardial apoptosis and Akt signaling pathways in a diabetic rat model. A cohort of forty male Wistar rats was systematically allocated into five distinct experimental groups: a control group, a diabetes mellitus (DM) group, a DM group receiving taurine (DM/T), a DM group undergoing CARE (DM/CARE), and a DM group receiving both taurine and CARE (DM/T/CARE). The induction of diabetes was achieved through the administration of streptozotocin and nicotine. Taurine was administered at a dosage of 100 mg/kg on a daily basis, while the CARE protocol was executed at an intensity corresponding to 40–60% of the one-repetition maximum (1RM) for a duration of 8 weeks. The diabetic condition resulted in a significant elevation in blood glucose levels, alongside increased concentrations of caspase-3 and caspase-9, as well as a rise in apoptotic cardiomyocytes, concurrently with a decrease in Akt expression (*P* < 0.001). Both taurine and CARE interventions, when applied independently, mitigated apoptosis and enhanced Akt levels, with the combined intervention (DM/T/CARE) exhibiting the most pronounced effect (*P* < 0.05) (12) (Table 3). Another study determined the impact of taurine supplementation on exercise capacity among individuals diagnosed with heart failure (HF). A total of 29 participants exhibiting left ventricular ejection fraction (LVEF) <50% and classified as NYHA functional class II–III were recruited; 15 individuals were administered taurine (500 mg, three times daily) while 14 were given a placebo over a 2-week period. All subjects underwent exercise tolerance assessments both prior to and following the supplementation phase. Baseline attributes, encompassing LVEF, body mass index, exercise duration, metabolic equivalents (METS), and exercise distance, were found to be comparable across the two groups. Following the supplementation period, the cohort receiving taurine exhibited statistically significant enhancements in exercise duration, METS, and exercise distance (*P* < 0.0001 for all), while the placebo cohort did not demonstrate any notable alterations (58).

**Table 3:** Effects of taurine supplementation combined with exercise on metabolic, inflammatory, and functional outcomes in diabetes mellitus.

**Population and design**	**Intervention**	**Results**	**Study**
40 male Wistar rats, 5 groups (control, DM, DM + taurine, DM + CARE, DM + taurine + CARE)	Taurine 100 mg/kg BW, gavage, 6 days/week, 8 weeks; CARE 40–60% 1RM	Blood glucose ↑ in DM; Caspase-3/9 ↑ in DM; Akt ↓ in DM; Taurine + CARE → Blood glucose ↓; Caspase-3/9 ↓; Akt ↑ (greatest improvement in DM/T + CARE)	(12)
29 HF patients, LVEF <50%, NYHA class II–III; 15 taurine, 14 placebo	Taurine 500 mg, 3x/day for 2 weeks; exercise tolerance test	Exercise time ↑; METS ↑; Exercise distance ↑ in taurine group; Placebo → no change	(58)
30 male Wistar rats; 3 groups: diabetic exercise + taurine (*n* =10), diabetic control (*n* = 10), healthy control (n=10)	8 weeks combined aerobic + strength training 5 × /week + taurine 1% in drinking water	BDNF ↑ in diabetic exercise + taurine; CRP ↓ in diabetic exercise + taurine; IL-6 → no change	(59)
40 middle-aged females with type 2 diabetes; 4 groups: exercise, taurine, exercise + taurine, control	8 weeks TRX resistance training 3 × /week + taurine supplementation	HBA1C ↓ in exercise, taurine, and exercise + taurine groups; FBS ↓ in exercise group vs. exercise + taurine; control → no change	(60)
40 male Wistar rats; 4 groups: diabetic control, diabetic + training, diabetic + taurine, healthy control	8 weeks combined endurance + resistance exercise 5 × /week + taurine 1% in water	PI3K ↑ in diabetic training group; IL-1β ↑ in diabetic training group; AKT → no change; taurine alone → no change	(61)
Male Wistar rats; 5 groups: control, DM, DM + taurine, DM + exercise, DM + exercise + taurine	8 weeks alternating resistance/endurance + taurine 100 mg/kg, 6 × /week	Bax ↓ in taurine, exercise, taurine + exercise; Bcl2 ↑ in taurine, exercise, taurine + exercise; Bax/Bcl2 ratio ↓ in taurine + exercise; collagen deposition ↓ in all, most in taurine+exercise	(62)
40 male Wistar rats; 3 diabetic groups + 1 healthy control	8 weeks combined endurance-resistance exercise 5 × /week + taurine 1% in water	Osteocalcin ↑ in diabetic exercise group; Osteopontin → no change; Body weight & BMI ↑ in exercise group vs. supplement; Food intake ↓ in exercise group vs. supplement	(63)
50 male Wistar rats; 4 groups: exercise, taurine, exercise + taurine, control + 10 healthy	8 weeks resistance ladder + treadmill 75% VO_2_max 5 × /week + taurine 1% in water	LXR ↓ in supplement & exercise + supplement; Total cholesterol ↓ in exercise + supplement; Body weight & BMI ↓ in exercise, supplement & exercise + supplement	(30)
30 male Wistar rats; 3 groups: diabetic exercise + supplement, diabetic control, healthy control	8 weeks combined endurance-resistance training 5 × /week + taurine 1% in water	PI3K ↑ in exercise + supplement; IL-1β ↓ in exercise + supplement; AKT → no change	(64)
40 middle-aged women; 4 groups: training, supplement, training + supplement, control	8 weeks TRX resistance training 3 × /week + taurine 500 mg 3 × /day	HBA1C ↓ in supplement, exercise, and exercise + supplement; FBS ↓ in exercise vs. exercise + supplement	(28)
50 male Wistar rats; 5 groups: diabetic exercise, diabetic supplement, exercise + supplement, diabetic control, healthy control	8 weeks endurance-resistance training + taurine 1% in water	Aldosterone ↓, Renin ↓, Angiotensin ↓ in exercise, supplement, and exercise + supplement groups	(65)

↑ Increased, ↓ Decreased. DM, Diabetes Mellitus; CARE, Combined Aerobic and Resistance Exercise; HF, Heart Failure; LVEF, Left Ventricular Ejection Fraction; METS, Metabolic Equivalents; STZ, Streptozotocin; BDNF, Brain-Derived Neurotrophic Factor; CRP, C-Reactive Protein; IL-6, Interleukin-6; FBS, Fasting Blood Sugar; HBA1C, Glycosylated Hemoglobin; AKT, Protein Kinase B; PI3K, Phosphoinositide 3-Kinase; IL-1β, Interleukin-1 Beta; ERE, Endurance-Resistance Exercise; Bax, Bcl-2-associated X protein; Bcl2, B-cell lymphoma 2; BMI, Body Mass Index; LXR, Liver X Receptor; TRX, Total Resistance Exercise.

Pahlevani et al. (59) assessed the impact of concurrent aerobic and resistance training, alongside taurine supplementation, on the biomarkers BDNF, CRP, and IL-6 in a cohort of diabetic rats. A total of thirty adult male Wistar rats were systematically assigned into three distinct groups: diabetic exercise-supplementation (*n* = 10), diabetic control (*n* = 10), and healthy control (*n* = 10). The induction of diabetes was accomplished through the administration of streptozotocin at a dosage of 55 mg/kg via intraperitoneal injection. The diabetic exercise-supplementation group engaged in aerobic (75% VO-max) and resistance ladder-climbing exercises five times weekly over a duration of 8 weeks, concurrently receiving taurine in a 1% solution within their drinking water. Serum levels of BDNF exhibited a statistically significant elevation in the exercise-supplemented diabetic group when compared to the diabetic control group (*P* = 0.003), while CRP levels demonstrated a notable decrease (*P* = 0.008). Conversely, IL-6 levels did not display any statistically significant variations across the groups (*P* = 0.059). In addition, Samadpour Masouleh et al. (60) examined the implications of an 8-week regimen of TRX resistance training in conjunction with taurine supplementation on glycemic regulation among middle-aged women diagnosed with diabetes mellitus. A cohort of 40 physically inactive diabetic women (aged 40–60 years) was randomly allocated into four distinct groups: training, supplementation, combined training and supplementation, and a control group (*n* =1 0 per group). Fasting blood glucose (FBG) levels and HbA1c were assessed prior to and following the intervention period. The training protocol entailed three TRX sessions weekly over the duration of 8 weeks, with taurine supplementation administered simultaneously. The findings indicated a statistically significant reduction in HbA1c levels within the supplementation, training, and combined groups (*P* ≤ 0.027), while no significant alterations were observed in the control group. Furthermore, FBG levels exhibited a significant decline in the training group when compared to the combined group (*P* = 0.012).

Also, Rahmatollahi and Pourrahim Ghouroghchi (61) evaluated the implications of an 8-week regimen of synergistic endurance-resistance training and taurine supplementation on cardiac inflammation and glucose signaling pathways in diabetic male Wistar rats. A total of forty rats were systematically allocated into four distinct groups: a diabetic control group, a diabetic group subjected to combined training, a diabetic group receiving taurine supplementation, and a healthy control group (*n* = 10 per group). The induction of diabetes was accomplished through the administration of streptozotocin at a dosage of 55 mg/kg. The combined training regimen was conducted five times per week, while taurine supplementation was provided in the form of a 1% solution incorporated into the drinking water. The findings indicated that the combined training significantly elevated PI3K gene expression by 57% and serum IL-1β levels by 42% in comparison to the diabetic control group (*P* < 0.01), whereas taurine supplementation alone did not yield any statistically significant outcomes. AKT gene expression exhibited no notable variation among the different experimental groups. In addition, Sedaghat (62) shown the impact of taurine supplementation in conjunction with endurance-resistance exercise (ERE) on cardiac apoptosis and fibrosis in diabetic male Wistar rats. A total of forty rats were randomly allocated into five distinct groups: control, diabetes mellitus (DM), DM + taurine, DM + ERE, and DM + taurine + ERE. The induction of diabetes was accomplished through the administration of nicotinamide (100 mg/kg) and streptozotocin (55 mg/kg). Taurine (100 mg/kg) was administered orally six times per week over a duration of 8 weeks, with the exercise regimen alternating between resistance and endurance training. Cardiac tissues were subjected to analysis for the expression of Bax and Bcl2, as well as collagen deposition. Both taurine and ERE, when administered independently, significantly reduced Bax levels and enhanced Bcl2 expression in comparison to the DM group (*P* ≤ 0.05), whereas the synergistic application of both treatments notably normalized the Bax/Bcl2 ratio to levels observed in the control group (*P* ≤ 0.001). Furthermore, collagen deposition was significantly diminished in the group receiving the combined intervention. Akbari Vargsaran and Pourrahim Ghoroghchi (63) examined the ramifications of an 8-week regimen of combined endurance-resistance training (ERT) alongside taurine supplementation on the concentrations of osteocalcin and osteopontin in diabetic male Wistar rats. A total of forty rats (weighing 250–300 g, aged 6 weeks) were systematically allocated into four distinct groups: diabetic control, diabetic exercise, diabetic taurine supplementation, and healthy control. The induction of diabetes was achieved through the administration of streptozotocin (55 mg/kg). The exercise intervention was conducted five times per week over the duration of 8 weeks, while taurine supplementation was provided daily as a 1% solution in the drinking water. The findings indicated a statistically significant elevation in osteocalcin levels within the diabetic exercise cohort when juxtaposed with the diabetic control group (*P* = 0.018), whereas osteopontin levels exhibited a significant increase in the diabetic control group relative to the healthy control group (*P* = 0.003), with no notable differences observed among the other groups. Furthermore, body weight and body mass index (BMI) exhibited an increase in the diabetic exercise group in comparison to the diabetic supplementation group, accompanied by a reduction in food intake (*P* = 0.03).

Ghouroghchi and Orange (30) assessed the ramifications of an 8-week regimen of integrated resistance-endurance training (ERT) coupled with taurine supplementation on the expression levels of LXR and serum total cholesterol concentrations in diabetic male Wistar rats. A cohort of fifty rats (weighing between 200 and 220 g and aged 6 weeks) was systematically allocated into five distinct groups: healthy control, diabetic control, exercise, taurine supplementation, and a combined exercise plus taurine intervention. The induction of diabetes was achieved through the administration of streptozotocin (55 mg/kg, i.p.). The resistance training protocol entailed ladder climbing executed 15 times, whereas the endurance training component was conducted on a treadmill at an intensity of 75% VO_2_max, occurring five times per week. Taurine supplementation was provided in the form of a 1% solution incorporated into the drinking water. Following the completion of the 8-week intervention, a statistically significant reduction in LXR expression was observed in both the taurine and exercise plus taurine groups when juxtaposed with the control group (*P* = 0.0001), while a notable decrease in total cholesterol was recorded exclusively in the combined exercise plus taurine group (*P* = 0.027). Furthermore, body weight and Body Mass Index (BMI) exhibited a significant reduction in the exercise, taurine, and combined groups relative to the diabetic control group. Furthermore, Rahmatollahi and Pourrahim Ghouroghchi (64) examined the implications of an 8-week regimen of integrated physical activity and taurine supplementation on cardiac inflammatory biomarkers in diabetic male Wistar rats. A total of 30 rats (weighing between 250 and 300 g, aged 6 weeks) were randomly allocated into three distinct groups: diabetic control, diabetic exercise with taurine supplementation, and healthy control. The induction of diabetes was achieved via administration of streptozotocin (55 mg/kg, i.p.), with blood glucose levels exceeding 250 mg/dL serving as confirmation of the diabetic state. The exercise regimen incorporated both endurance and resistance training, executed five times per week, while taurine was administered in the form of a 1% solution within the drinking water. Post-intervention analysis revealed a significant enhancement in PI3K gene expression within the diabetic exercise plus taurine group when compared to the diabetic control (P=0.001), alongside a noteworthy reduction in serum IL-1β levels (*P* = 0.001). No statistically significant alterations were detected in AKT gene expression.

**Figure 3:**
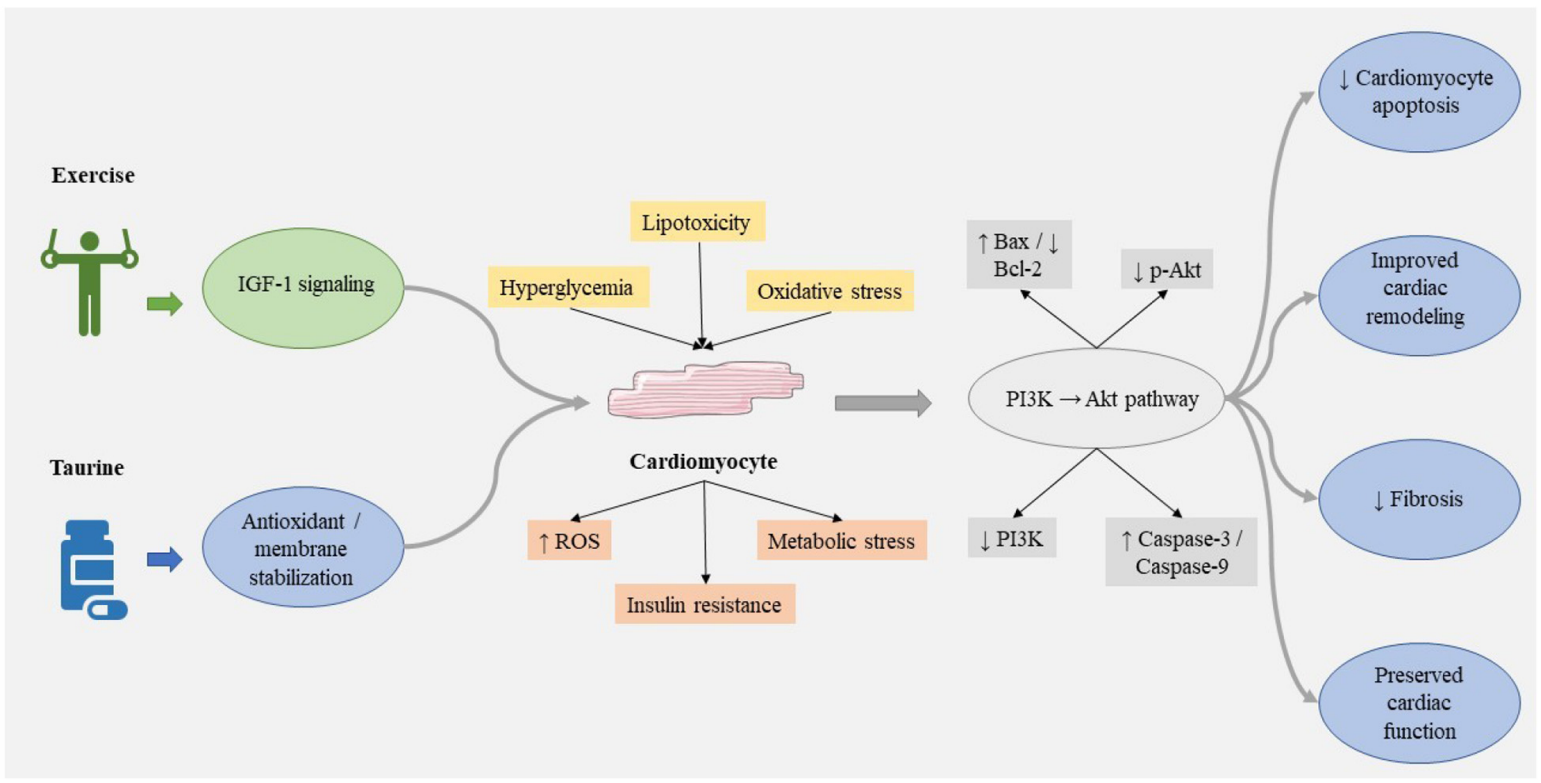
Proposed mechanisms by which taurine supplementation and physical exercise modulate PI3K/Akt signaling and influence cardiac remodeling in diabetes. Schematic representation illustrating potential additive or complementary effects of taurine supplementation and combined aerobic–resistance exercise on cardiomyocyte survival signaling and structural remodeling in diabetic conditions. Diabetes-associated pathological features include metabolic stress (↑), oxidative damage (↑ reactive oxygen species), impaired insulin signaling (↓), suppression of the PI3K/Akt survival pathway (↓), and activation of pro-apoptotic cascades, including caspase-3 and caspase-9 (↑), contributing to cardiomyocyte apoptosis and adverse cardiac remodeling. Taurine and exercise are depicted as converging modulators that may promote reactivation of PI3K/Akt signaling (↑), enhance anti-apoptotic protein expression such as Bcl-2 (↑), and reduce mitochondrial-dependent apoptotic signaling (↓). These pathway interactions are associated with decreased cardiomyocyte loss (↓ apoptosis), attenuation of fibrotic remodeling (↓), and preservation of myocardial structure and functional integrity (↑). Solid arrows indicate pathways supported by experimental evidence, whereas dashed arrows denote proposed or emerging mechanisms inferred primarily from preclinical studies. Upward arrows (↑) represent activation or increased activity/expression, while downward arrows (↓) indicate inhibition or reduction. Although combined effects suggest cardioprotective potential, direct evidence demonstrating mechanistic synergy in humans remains limited.

Masouleh et al. (28) evaluated the impact of an 8-week regimen of TRX resistance training in conjunction with taurine supplementation on glycemic metrics among middle-aged women diagnosed with diabetes mellitus. A cohort of 40 participants aged between 40 and 60 years, all exhibiting inactive diabetes, was randomly allocated to one of four distinct groups: exercise-only, taurine supplementation-only, combined exercise + taurine, and a control group. The TRX resistance training protocol was executed three times weekly over the duration of 8 weeks, while taurine supplementation was administered on a daily basis. Fasting blood glucose (FBS) and HbA1c levels were assessed both prior to and following the intervention. The findings indicated significant reductions in HbA1c levels within the supplementation (*P* = 0.027), exercise (*P* = 0.001), and combined exercise + supplementation groups (*P* = 0.001) in comparison to baseline measurements, whereas no notable alterations were documented within the control group. Furthermore, FBS levels were observed to be significantly lower in the exercise group compared to the combined group (*P* = 0.012). Rajaei Ghasem Gheshlaghi et al. (65) documented the additive effects of an 8-week regimen of endurance-resistance training in conjunction with taurine supplementation on the renin-angiotensin-aldosterone system in diabetic male Wistar rats. A total of 40 male rats, which were subjected to streptozotocin-induced diabetes, were randomly allocated into five distinct groups: diabetic control, diabetic exercise, diabetic taurine supplementation, diabetic exercise combined with taurine, and healthy control. The implementation of endurance-resistance training and the administration of taurine (1% solution in drinking water) were conducted over the span of 8 weeks. The findings revealed statistically significant decreases in aldosterone, renin, and angiotensin concentrations in the exercise, taurine, and combined exercise + taurine groups when contrasted with diabetic controls (*P* = 0.0001). Overall, taurine supplementation, in conjunction with both aerobic and resistance training, manifests a diverse array of molecular influences in the context of diabetes. These therapeutic strategies mitigate cardiomyocyte apoptosis by diminishing the Bax/Bcl-2 ratio and inhibiting the activation of caspase-3/9, while simultaneously amplifying anti-apoptotic signaling through the PI3K/Akt pathways, thereby facilitating cardiac remodeling and attenuating fibrosis. In parallel, taurine and physical exercise elevate PI3K expression and adjust inflammatory biomarkers, notably by reducing levels of IL-1β and CRP, thereby stabilizing systemic inflammatory responses. The upregulation of neurotrophic factors, particularly brain-derived neurotrophic factor (BDNF), contributes to enhanced resilience of both cardiac and neuronal tissues. The metabolic advantages encompass improved glycemic regulation, a decrease in fasting blood glucose and HbA1c levels, augmented insulin sensitivity, and the modulation of lipid profiles through the reduction of total cholesterol and LDL cholesterol. Furthermore, taurine, when combined with exercise, exerts regulatory effects on renal and cardiovascular hormones such as aldosterone, renin, and angiotensin, while also impacting bone metabolism through the elevation of osteocalcin levels. In summary, taurine operates complementary with exercise to mitigate apoptosis, inflammation, metabolic disturbances, and organ dysfunction associated with diabetes (Figure 3).

Across preclinical and clinical studies in diabetes, taurine supplementation combined with exercise generally attenuates cardiac apoptosis, improves glycemic regulation, and modulates inflammatory signaling; however, the consistency and mechanism of interaction between the two interventions vary substantially across models. Several diabetic rodent studies report that combined aerobic and resistance training with taurine produces greater reductions in apoptotic markers and more pronounced activation of PI3K-related survival signaling than either intervention alone, supporting a cooperative cardioprotective effect under conditions of metabolic stress. Nevertheless, not all mechanistic studies confirm additive pathway activation: some investigations demonstrate improvements in PI3K expression or inflammatory markers with exercise, while taurine alone produces limited or no independent signaling changes, suggesting that exercise may function as the dominant stimulus for pathway modulation in certain contexts.

Clinical investigations in humans consistently report improvements in functional outcomes such as exercise capacity, glycemic control, and inflammatory indices following taurine supplementation and/or training interventions, yet direct confirmation of cardiac signaling mechanisms is largely absent due to limited access to tissue-level endpoints. Consequently, translational alignment between molecular findings in animal models and functional outcomes in human populations remains incomplete.

Methodological heterogeneity further contributes to divergent findings. Animal studies typically employ controlled disease induction, body-weight-adjusted taurine dosing, and combined training protocols, whereas human trials vary in exercise modality, supplementation duration, and participant metabolic severity. Differences in diabetes induction models, intervention intensity, and outcome measures also influence whether additive, independent, or overlapping effects are observed.

Collectively, current evidence supports a context-dependent interaction in which taurine may enhance exercise-mediated cardiometabolic protection, particularly under conditions of severe metabolic dysfunction. However, it remains unresolved whether taurine directly amplifies PI3K/Akt signaling in humans, whether observed benefits reflect parallel rather than synergistic mechanisms, and which dosing or training parameters optimize combined therapeutic effects. These uncertainties highlight the need for mechanistically integrated clinical studies linking pathway-level adaptations with functional cardiovascular outcomes in diabetes.

### 6.4 Preclinical and clinical evidence on mTOR pathway

Emerging evidence suggests that taurine exerts broad metabolic regulatory effects across multiple physiological systems, particularly under conditions of metabolic dysfunction such as diabetes. Experimental and clinical studies have indicated that taurine supplementation may contribute to improved glycemic regulation through several mechanisms, including modulation of glucagon activity, stabilization of blood glucose levels, enhancement of insulin secretion, and attenuation of insulin resistance. Additionally, taurine has been associated with reductions in hyperglycemia, partly through the modulation of advanced glycation end-product formation, which represents a key pathological process contributing to diabetic complications. Beyond glycemic control, taurine has demonstrated protective effects against oxidative stress, a central driver of metabolic and cellular dysfunction in diabetes (66).

The pleiotropic actions of taurine extend to multiple organ systems affected by metabolic disease. Neuroprotective effects have been reported, including attenuation of oxidative stress within the brain, promotion of hormone regulation, and protection against diabetic neuropathy. Similarly, taurine has been associated with protective outcomes in diabetic retinopathy and nephropathy, suggesting systemic benefits linked to improved metabolic homeostasis and reduced inflammatory burden. In peripheral tissues, taurine has shown efficacy in mitigating diabetic hepatotoxicity, vascular dysfunction, and cardiac injury, further highlighting its potential as a multi-target metabolic modulator. Many of these effects are thought to be mediated through improvements in mitochondrial function, redox balance, and cellular stress responses (66).

Experimental evidence supporting a mechanistic link between taurine and mTOR signaling has been demonstrated in studies investigating metabolic dysfunction induced by environmental stressors. In a study examining low-level inorganic arsenic exposure, taurine supplementation was shown to improve insulin resistance and restore impaired glucose metabolism through activation of the PPARγ-mTORC2 signaling axis (67). Arsenic exposure reduced glycolytic capacity, increased gluconeogenic signaling, and suppressed hepatic PPARγ and mTORC2 expression, contributing to metabolic dysregulation. Taurine administration reversed these alterations, promoting mTORC2 activation and improving insulin sensitivity, while concurrently inhibiting excessive hepatic autophagy (67). These findings suggest that taurine may exert metabolic benefits by modulating nutrient-sensing pathways that integrate insulin signaling and cellular stress responses. Importantly, the involvement of mTORC2 highlights a potential mechanism through which taurine influences metabolic flexibility, as mTORC2 is closely associated with Akt signaling, glucose homeostasis, and lipid metabolism. Although derived primarily from preclinical models, these results provide mechanistic support for the hypothesis that taurine-mediated regulation of mTOR signaling contributes to improved metabolic adaptation under pathological conditions, including diabetes and toxin-induced metabolic stress (67).

Clinical evidence supporting the metabolic and anti-inflammatory effects of taurine has been demonstrated in a randomized, double-blind, placebo-controlled trial conducted in patients with type 2 diabetes mellitus (6). In this study, taurine supplementation significantly improved oxidative stress indices by increasing antioxidant enzyme activities, including superoxide dismutase (SOD) and catalase (CAT), while reducing lipid peroxidation markers such as malondialdehyde (MDA). Additionally, taurine intake was associated with reductions in key inflammatory biomarkers, including high-sensitivity C-reactive protein (hs-CRP) and tumor necrosis factor-α (TNF-α), suggesting a systemic anti-inflammatory effect in human metabolic disease (6). These findings support the hypothesis that taurine contributes to improved metabolic homeostasis by attenuating oxidative stress and chronic low-grade inflammation, two processes closely linked to impaired insulin signaling and metabolic dysfunction. Although direct measurement of mTOR signaling was not performed, the observed improvements in redox balance and inflammatory pathways are consistent with mechanisms known to influence mTOR activity, particularly through modulation of upstream regulators such as insulin signaling and cellular stress responses (6). Therefore, this clinical trial provides translational evidence that taurine may indirectly support metabolic regulation via pathways converging on mTOR-dependent signaling networks, highlighting potential relevance for therapeutic strategies targeting metabolic flexibility in diabetes (6).

Despite growing evidence supporting independent roles for taurine supplementation and physical exercise in modulating metabolic signaling pathways, including those associated with mTOR regulation, direct evidence demonstrating synergistic interactions between taurine and exercise on mTOR signaling remains limited. While both interventions appear to influence upstream regulators such as insulin signaling, oxidative stress responses, and cellular energy sensing, current literature primarily describes their effects in isolation rather than within combined experimental paradigms. Consequently, it cannot be concluded that taurine and exercise exert synergistic modulation of mTOR-dependent pathways based on existing data. The absence of studies specifically examining combined interventions highlights an important gap in the literature and underscores the need for future mechanistic investigations designed to evaluate potential additive, complementary, or synergistic effects under controlled conditions. Further research integrating preclinical and clinical approaches will be essential to clarify whether coordinated modulation of mTOR signaling contributes to enhanced metabolic adaptation when taurine supplementation is combined with structured exercise interventions.

## 7 Integration: systems-level effects across obesity, aging, and diabetes

### 7.1 Cross-talk between adipose, muscle, brain, and heart

Taurine supplementation, particularly when synergistically administered alongside physical exercise, manifests pleiotropic effects across a multitude of organ systems, thereby underscoring the systemic nature of its advantages in the contexts of metabolic disorders and aging. Within adipose tissue, taurine enhances lipid metabolism, augments thermogenic potential, and regulates adipokine release, which collectively contributes to the amelioration of systemic insulin sensitivity and metabolic adaptability (68, 69). The skeletal muscle experiences notable benefits from taurine-mediated enhancements in mitochondrial functionality, as well as improvements in both aerobic and anaerobic performance, and anti-inflammatory signaling, all of which foster exercise tolerance and elevate energy expenditure (17). In the realm of the central nervous system, taurine demonstrates neuroprotective properties by attenuating neuroinflammation, reinstating BDNF signaling pathways, and alleviating cognitive decline associated with aging or diabetes (70, 71). The myocardium exhibits a pronounced sensitivity to the additive effects of taurine and physical exercise, wherein the augmentation of PI3K/Akt signaling pathways, a reduction in apoptosis (as indicated by the Bax/Bcl2 ratio), and a diminution in collagen deposition collectively enhance cardiac remodeling and functionality in the context of metabolic stress. These observations imply that taurine functions as a systemic modulator, facilitating intricate intercommunication among adipose tissue, skeletal muscle, the central nervous system, and cardiac tissue to mitigate the impacts of metabolic and age-associated impairments.

### 7.2 Translational implications

The systemic and multi-organ ramifications of taurine, when coupled with structured exercise interventions, yield promising translational prospects for the management of obesity, diabetes, and age-related metabolic deterioration. Within clinical settings, taurine supplementation may augment exercise capacity and cardiovascular functionality in individuals suffering from heart failure or diabetes mellitus, while concurrently enhancing glycemic regulation and neurocognitive outcomes (8, 42, 72). Significantly, taurine's modulation of inflammatory and apoptotic pathways across various organ systems positions it as a cost-effective, non-invasive complement to traditional lifestyle modifications and pharmacological treatments. Prospective human studies employing multi-omics and imaging methodologies are essential to quantitatively assess taurine-induced tissue plasticity, refine dosing strategies, and investigate long-term advantages in metabolic and neurodegenerative disorders.

## 8 Future directions and clinical perspectives

Although an increasing body of evidence elucidates the additive advantages of taurine supplementation in conjunction with exercise on the plasticity of adipose tissue, metabolic adaptability, neuroinflammaging, and cardiac remodeling, significant deficiencies persist that hinder the clinical application of these findings. The majority of mechanistic understandings are derived from short-duration preclinical investigations utilizing rodent models, which may not adequately replicate human physiological responses, especially in aging or diabetic demographics. The ideal dosage, timing, and formulation of taurine for various exercise modalities remain inadequately characterized. Furthermore, the interplay between taurine and factors such as sex, age, baseline metabolic conditions, and comorbidities including obesity, diabetes mellitus, and cardiovascular diseases remains largely unexamined. Longitudinal investigations assessing safety, specifically regarding chronic supplementation in older populations, are notably limited. Analyses driven by biomarkers for stratification and the differentiation between responders and non-responders are almost entirely absent, complicating the customization of interventions to align with individual metabolic and cognitive profiles. Ultimately, research that integrates multi-omics methodologies with functional outcomes could yield the mechanistic insights that are presently lacking, thereby constraining the precision of application. Taurine supplementation, when synergistically integrated with structured physical activity, presents significant potential as a non-pharmacological, multi-faceted intervention aimed at alleviating metabolic dysfunction, neurocognitive deterioration, and cardiac remodeling. Clinically, this approach may augment the efficacy of exercise in older adults, individuals experiencing obesity, and patients diagnosed with diabetes mellitus by enhancing glycemic regulation, insulin sensitivity, mitochondrial functionality, and cardiovascular robustness. Additionally, it may exert an influence on systemic and neuroinflammatory processes, thereby fostering cognitive health throughout the aging process. Future investigations should prioritize large-scale, randomized controlled trials encompassing diverse human populations, incorporating functional outcomes, molecular biomarkers, and extended follow-up periods to evaluate both efficacy and safety. Tailored dosing strategies informed by individual metabolic and inflammatory profiles could further refine and enhance therapeutic outcomes. Ultimately, the combination of taurine and exercise could represent a cost-effective, holistic approach to prolonging healthspan, diminishing age- and obesity-related morbidities, and bolstering cardiovascular and cognitive resilience throughout the human lifespan.

## 9 Conclusion

**Figure 4:**
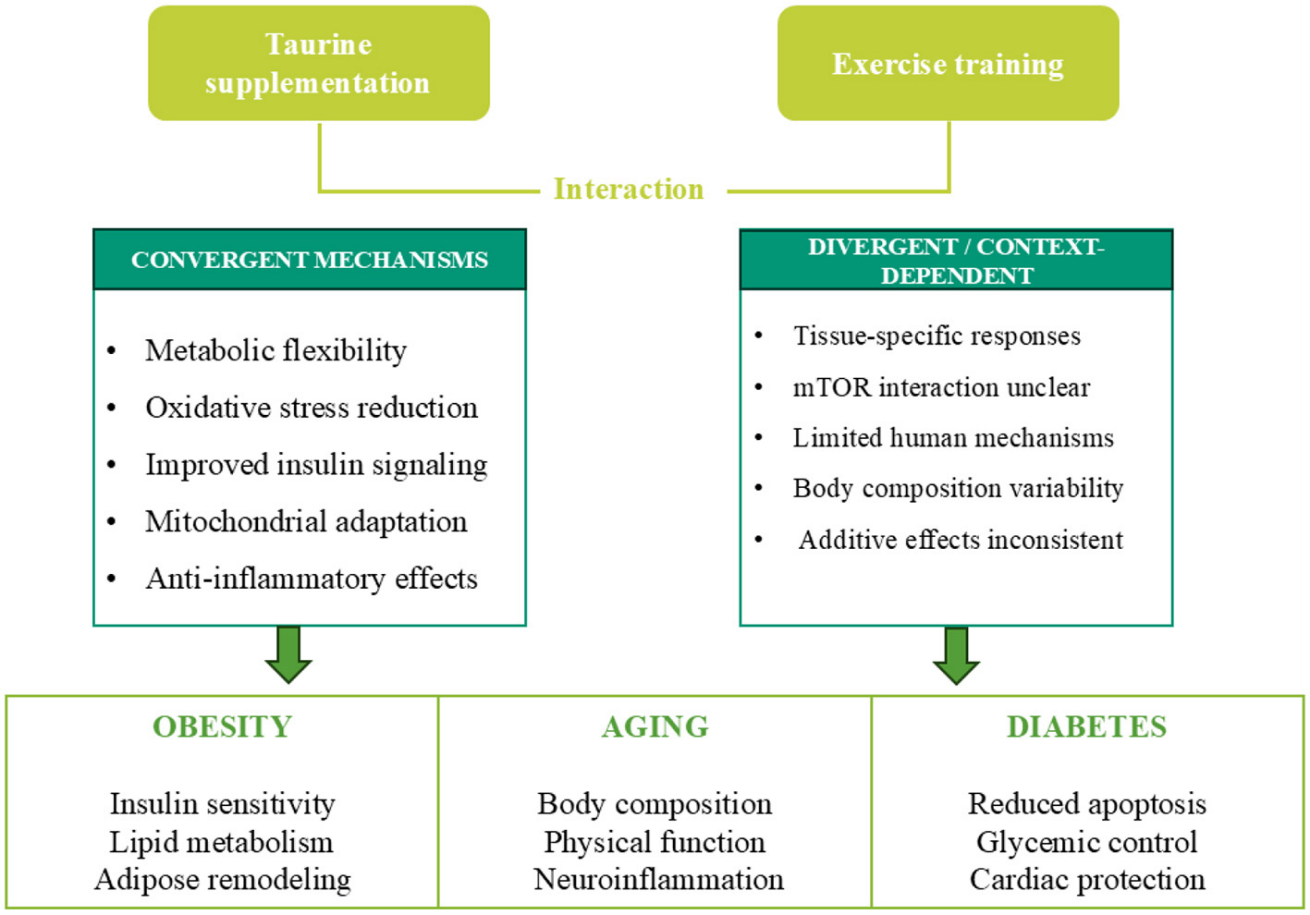
Convergent molecular and physiological mechanisms of taurine supplementation combined with exercise in obesity, aging, and diabetes. This schematic summarizes convergent and divergent findings across studies examining taurine supplementation combined with exercise in obesity, aging, and diabetes. Evidence from both preclinical and clinical investigations indicates consistent activation of shared biological mechanisms, including attenuation of oxidative stress, modulation of inflammatory signaling, improvement of mitochondrial function, and enhancement of metabolic flexibility. These convergent pathways are associated with beneficial physiological adaptations across adipose, cardiovascular, and neurofunctional domains. However, several outcomes remain context-dependent and exhibit substantial heterogeneity across studies. Inconsistent findings include variability in additive vs. non-additive effects of combined interventions, differential responses in body composition and functional outcomes, limited direct evidence for synergistic regulation of mTOR signaling, and incomplete translational alignment between molecular mechanisms and clinical endpoints in humans. Methodological variability in species, intervention design, dosage, exercise modality, and participant metabolic status likely contributes to these divergent observations.

Taurine supplementation, particularly when integrated with structured physical activity, emerges as a complex intervention capable of influencing critical physiological and molecular pathways pertinent to obesity, aging, and diabetes (Figure 4). Empirical evidence derived from both preclinical and clinical investigations indicates that this approach enhances adipose tissue plasticity, augments metabolic flexibility, mitigates systemic and neuroinflammation, bolsters cognitive performance, and fosters advantageous cardiac remodeling via PI3K/Akt signaling and apoptosis modulation. These complementary outcomes underscore taurine's distinctive function as a systems-level modulator that augments the well-documented advantages of exercise. Despite the promising findings, there exist considerable translational disparities, particularly concerning the optimal dosage, duration, and the effects of individual heterogeneity, encompassing factors such as age, sex, comorbidities, and initial metabolic health status. Subsequent research endeavors that incorporate longitudinal studies, multi-omics methodologies, and biomarker-oriented strategies will be essential for the enhancement of personalized intervention frameworks. In summary, the combination of taurine with physical exercise constitutes a safe, economically viable, and non-pharmacological intervention with extensive therapeutic implications. In addition to conventional metrics such as weight reduction and glycemic regulation, this method addresses systemic metabolic health, cardiovascular robustness, and neurocognitive safeguarding, thereby providing a comprehensive paradigm to enhance healthspan and quality of life in at-risk populations. These findings lay the groundwork for prospective clinical applications and precision lifestyle interventions aimed at obesity, aging, and diabetes.
